# Pollution Status, Ecological Risks, and Potential Sources of Metals in the Middle and Lower Reaches of the Lianjiang River Basin, Guangdong Province, China

**DOI:** 10.3390/toxics13100840

**Published:** 2025-10-01

**Authors:** Yongzhong Lai, Le Li, Xianbing Huang, Guoyong Lu, Fengqin Pan, Wenhua Liu

**Affiliations:** 1 Provincial Key Laboratory of Marine Biotechnology, Institute of Marine Science, Shantou University, Shantou 515063, China; whliu@stu.edu.cn; 2 Guangdong Shantou Ecological and Environment Monitoring Center Station, Shantou 515041, China; 13790848334@163.com (L.L.); 13414084791@163.com (X.H.); stlugy@163.com (G.L.); 15019708363@163.com (F.P.)

**Keywords:** Lianjiang River Basin, surficial channel sediment, heavy metals, source identification, risk assessment, integrated governance

## Abstract

Human activities have led to severe aquatic pollution and significant concerns about the ecological health of the Lianjiang River Basin (LRB). These concerns resulted in the implementation of comprehensive policies and treatments to improve the sediment and water quality. Herein, we explore the concentrations, sources, and degree of metal contamination in filtered water (FW), suspended solids (SSs), and surficial channel sediments (SCSs) in streams of the LRB. Calculated enrichment factors, an ecological risk index, and a principal component analysis were employed to understand the degree of elemental contamination, ecological risks, and their potential sources. Elements (e.g., Hg, Cd, Sn, Sb, Cu, and Mo) were mainly detected in FW, SSs, and SCSs in the Bergang, Hucheng, Xiashan, and Zhonggang rivers, and the mainstream of the LR. Four potential anthropogenic sources were identified, including electronic waste recycling (e.g., Cu, Sb, Pb, and Ni), mixed pollution (e.g., Se, Zn, Mn, and Mo), metal processing (e.g., Hg, Cr, Sn, and Cd), and battery manufacturing and recycling (e.g., Co, Ni, and Mn). Overall, Sn, Sb, Hg, Cu, and Cd were enriched by 37.5–79.2% and 34.8–91.3% at the SS and SCS sites, respectively. Mercury, Cd, Sn, Sb, Cu, and Mo posed the most risk both in the SSs and SCSs. Overall, the SS and SCS samples from the LRB remain severely contaminated with metals after recent environmental remediation. The implementation of pollution source control, sewage interception, and dredging operations should be further enhanced.

## 1. Introduction

Agricultural modernization, industrialization, and urbanization have resulted in the pollution of fresh waters on a global scale, leading to significant water resource concerns [1]. In fact, 70% of freshwater in developing economies is being gradually polluted [2]. The primary sources of these pollutants include industrial activities, metal mining, agricultural runoff (e.g., chemical fertilizers and pesticides), and atmospheric deposition [3].

In aquatic ecosystems, most hydrophobic pollutants, such as heavy metals (HMs), are deposited and enriched in aquatic sediments, particularly surficial channel sediments (SCSs) [4,5,6,7]. When the external geochemical conditions change, the SCSs may release HMs into the water and, therefore, act as a secondary source [8,9]. Thus, HMs in SCSs pose potential ecological risks [10,11], affecting everything from lower trophic organisms (e.g., benthos) to humans as they move through the food chain [12]. To enhance the health of the riverine ecosystem, it is essential to comprehend the characteristics of HMs in SCSs and determine their potential sources [13]. Sediment-associated HM contamination has become a topic of increasing concern since the 1980s (reviewed by [2,6,7,13,14,15,16]), and numerous studies have been carried out to identify the sources, transport, and enrichment of pollutants in SCSs [17,18].

The Lianjiang River Basin (LRB), in Guangdong Province, China, possesses several large urban areas including most of Puning in Jieyang City, Chaoyang District (CYD), and the Chaonan District (CND) of Shantou City. The Lianjiang River supplies the majority of their domestic and industrial water supplies. Unfortunately, however, the water quality within the basin has deteriorated significantly as the result of the combined effects of scarce precipitation and (1) intensified human discharges of illegal and/or unregulated electronic waste (E-waste) recycling in Guiyu Town, (2) textile printing and dyeing effluent, (3) the direct discharge of domestic and agricultural sewage, and (4) the disorderly stacking of domestic garbage and industrial waste. Water quality degradation has increasingly drawn public attention, particularly because of its impacts on cities along the LRB. As a result, the LRB has consistently been a focal point of environmental research and multiple studies have shown that the mainstream of the LRB is polluted [19,20,21], as are its major tributary rivers in Guiyu Town, such as the Bergang River and Old Lianjiang River [19,21,22,23,24,25,26,27], where serious HM pollution has been identified. The primary contaminant source reported to date is associated with E-waste recycling. The importance of other sources, other areas with severe metal pollution, and HM pollution in suspended solids (SSs) in the surface water in the LRB have largely been ignored.

Between June 2018 and December 2020, large-scale environmental improvement measures were implemented on the LRB, including the closure of printing and dyeing enterprises, the separation of rainwater and sewage systems, the construction of sewage treatment plants, waste incineration facilities, and sluice gates, the stabilization of river embankments, the creation of specialized industrial parks for the printing and dyeing sector, and the dredging of tributaries. Data on the status and sources of heavy metals (e.g., Sb, Sn, etc.) within the middle and downstream reaches of the LRB, particularly after environmental improvements, remain limited. As a result, there is a need to accurately evaluate the impacts of toxic elements on the aquatic ecosystem of the LRB following the implementation of the remediation measures. The primary objectives of this study are to assess the degree of water and SCS contamination and the primary contaminant sources, with respect to phosphorus (P), HMs, and organic matter (OM) following the remediation of the LRB. Inherent in these objectives is the need to (1) investigate the spatial distribution of P and HMs in filtered water (FW), SSs, and SCSs from the mainstream and primary tributaries of the LRB; (2) identify potential anthropogenic sources within the basin; (3) assess the potential ecological risks associated with metals in SSs and SCSs; (4) evaluate the effectiveness of pollution control measures implemented in the mainstream and primary tributaries of the LRB; and (5) conduct an in-depth examination of the root causes contributing to the persistent severe pollution situation and to formulate basin management strategies specifically designed to further reduce pollution and risk levels. This study provides valuable insights into the integrated management of P, metal, and OM pollution in the mainstream and primary tributaries of the LRB, and serves as a scientific reference for a human health risk assessment of the studied contaminants. It also offers practical guidance for the development of further treatment strategies in the region.

## 2. Materials and Methods

### 2.1. Study Area

**Figure 1:**
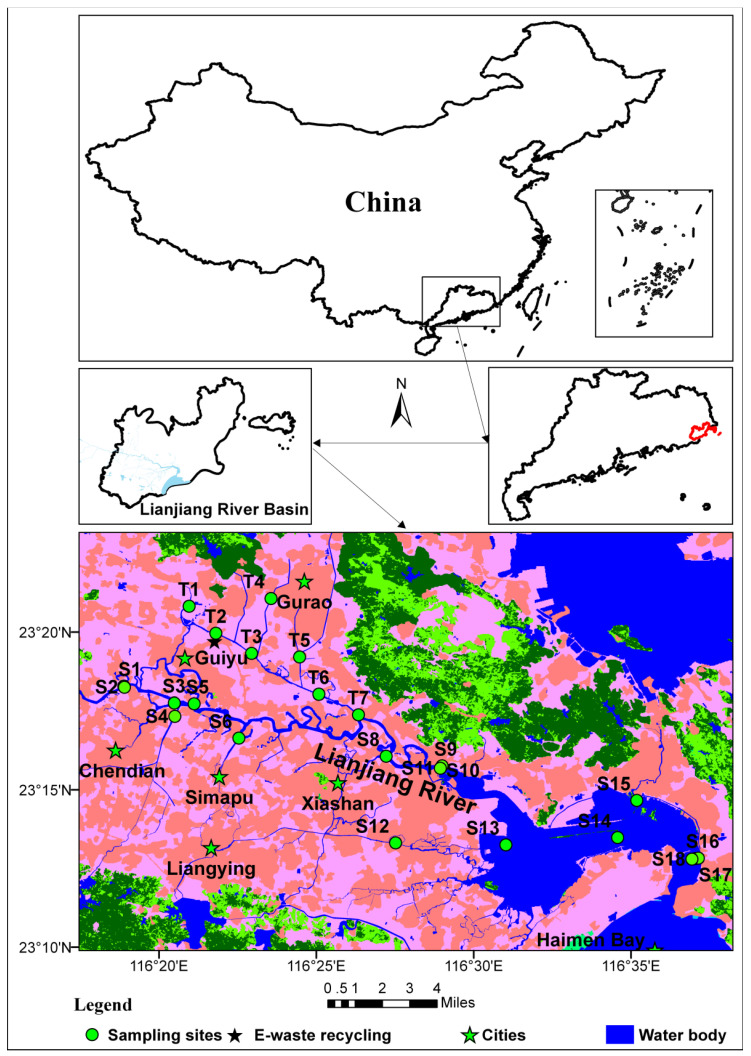
Location map of the study area showing sampling sites. Maps at bottom of figure provide more detailed information of the sampling locations along the Lianjing River.

The study area is situated in the eastern part of Guangdong Province, China (Figure 1). LR originates in the Wufeng Mountains of Puning City of Jieyang City and flows from west to east through Puning City, Chaonan, and the Chaoyang District of Shantou City to the South China Sea at Haimen Bay (Shantou City). The LRB covers an area of 1346.6 km^2^ [28]. It is the sole local sewage-receiving water body in the area. The LRB has limited clean water resources other than the LR and its tributaries, in part because its average annual precipitation is only about 7700 mm [28]. Thus, the LRB is crucial for socioeconomic development, the regional ecological environment, and the livelihoods of the majority of the population in Puning City, the Chaoyang District, and the Chaonan District, which have a combined population of over 4.3 million people [29].

### 2.2. Sampling and Analytical Procedures

Samples were collected from a total of 24 sites (Figure 1, Table S1), encompassing the mainstream and tributaries of the LR. Water samples were filtered through 10 cm diameter filters with 0.45 μm in pores (Shanghai Xin Ya Purification Equipment Co., Ltd., Shanghai, China) as promptly as possible. Both SSs on the filter and FW samples were collected. The SSs were collected from surface water samples via filtration and the sample volume was accurately recorded. The filters containing SSs were subjected to acid digestion according to the standardized protocol described below. Total phosphorus (P) in FW samples was analyzed following the procedure outlined in GB/T 11893-1989 [30]. Phosphorus in SSs was determined by digesting the SSs using the International Organization for Standardization procedure ISO 14869-1-2001 [31], followed by the analysis of aliquots by Inductively Coupled Plasma Optical Emission Spectrometry (ICP-OES, Optima 8000, PerkinElmer Inc., Waltham, MA, USA). Mercury (Hg), arsenic (As), and selenium (Se) in SS and FW samples were determined by Atomic Fluorescence Spectrometry (AFS, BAF-200C, Baode Instruments Ltd., Beijing, China) in accordance with HJ 680-2013 [32] and HJ 694-2014 [33].

At each site, about 2 kg of SCSs (0–10 cm) were collected using a grapple and subsequently placed in plastic bags. Samples were transported to the laboratory on the same day and cleaned of plant debris and stones. After air-drying indoors, samples were ground and passed through a 100-mesh nylon sieve. The pH of the SCSs was determined at a SCS:water ratio of 1:3 (*w*/*w*). Following thorough stirring for 1 min, the mixture was allowed to stand undisturbed for 30 min. Thereafter, the pH of the sample suspension was measured using a calibrated pH meter (pHTestr30, Eutech Instruments Pte Ltd., Singapore, Singapore). The OM content (in g/kg) of the SCSs was quantified using the NY/T 1121.6-2006 method [34].

Total Hg in the SCSs was measured with a direct mercury analyzer (DMA, BDHg-60, Baode Instruments Ltd., Beijing, China) in accordance with the HJ 923-2017 [35] method, whereas HJ 680-2013 [32] was used for analysis of total As and Se. Approximately 0.5 g of SCSs with a particle size of 100 mesh was accurately weighed and transferred into a pre-cleaned 50 mL glass tube that had been soaked in 10% (*v*/*v*) HNO_3_ solution for at least 72 h and subsequently rinsed thoroughly with ultrapure water (resistivity: 18.2 MΩ·cm). Initially, approximately 2 mL of ultrapure water was introduced into the tube, followed by the addition of 6 mL of HCl (ultrapure grade, Xilong Scientific Co., Ltd., Shantou, China) and 2 mL of HNO_3_ (ultrapure grade, Xilong Scientific Co., Ltd., Shantou, China). The tube was placed on the sample rack of a graphite digestion block and a glass funnel was fitted over the tube opening. Reflux digestion was then carried out at 120 °C for 120 min. After digestion, the digestate was allowed to cool to room temperature. The funnel was rinsed with ultrapure water and the rinseate was transferred back into the tube. The solution was then diluted to the mark with ultrapure water. The tube was capped and the mixture was gently mixed, followed by a settling period of 48 h. Subsequently, the supernatant was collected for the determination of total As and Se concentrations using Atomic Fluorescence Spectrometry (AFS, BAF-200C, Baode Instruments Ltd., Beijing, China).

The Cd in the SS and SCS samples was analyzed in accordance with GB/T 17141-1997 [36]. Approximately 0.1 g of sediment sample with a particle size of 100 mesh was accurately weighed and transferred into a pre-cleaned polytetrafluoroethylene (PTFE) digestion tube that had been soaked in 10% (*v*/*v*) HNO_3_ solution for at least 72 h and subsequently rinsed thoroughly with ultrapure water (resistivity: 18.2 MΩ·cm). Subsequently, 5 mL of HCl (ultrapure grade, Xilong Scientific Co., Ltd., Shantou, China) was added to the tube, and the mixture was heated at 120 °C until the volume was reduced to approximately 2 mL. Subsequently, 5 mL of HNO_3_ (ultrapure grade, Xilong Scientific Co., Ltd., Shantou, China), 1 mL of HF (analytical grade, Sinopharm Chemical Reagent Co., Ltd., Shanghai, China), and 2 mL of HClO_4_ (ultrapure grade, Tianjin Kemiou Chemical Reagent Co., Ltd., Tianjin, China) were added. A PTFE funnel was fitted onto the mouth of the tube and reflux digestion was performed at 120 °C for 4 h. After completion of the reflux step, the funnel was removed and heating was continued until the digestate became viscous. The tube was allowed to cool slightly, followed by rinsing the funnel and inner walls of the tube with ultrapure water. Then, 2.5 mL of HNO_3_ was added to dissolve the residue, and the resulting solution was quantitatively transferred to a pre-cleaned 50 mL glass tube that had been treated with 10% (*v*/*v*) HNO_3_ for no less than 72 h and extensively rinsed with ultrapure water. Subsequently, 3 mL of 5% (*w*/*v*) diammonium hydrogen phosphate solution (ultrapure grade, Tianjin Kemiou Chemical Reagent Co., Ltd., Tianjin, China) was added. After cooling to room temperature, the solution was diluted to the mark with ultrapure water. The tube was capped and the mixture was gently mixed, followed by a settling period of 48 h. Finally, the supernatant was collected for instrumental analysis.

The total concentrations of other elements in FW, SS, or SCS samples were prepared in accordance with HJ 700-2014 [37], HJ 776-2015 [38], and ISO 14869-1-2001 [31] using an automatic graphite digestion system. The concentrations of metals in the digests were examined by ICP-OES (e.g., P, Al, Fe, Ti, Mn, Cu, Zn, Cr, and Ni) and inductively coupled plasma mass spectrometry (ICP-MS, XSERIES II, Thermo Fisher Scientific Inc., Waltham, MA, USA) (e.g., Be, Co, Cd, Mo, Pb, Sb, and Sn) in accordance with U.S. EPA Method 6010D [39] and U.S. EPA Method 6020B [40]. All of the reported OM and metal concentrations in SSs and SCSs were normalized on a dry weight basis except for pH. The accuracy of sample analysis was evaluated based on the recoveries of reference samples within routine ranges of 75.0–130% for P, OM, and metals, with relative deviations among the results of parallel samples predominantly below 20%. Detailed sample collection and chemical analytical methods are given in Text S1.

### 2.3. Data Processing and Statistical Analyses

#### 2.3.1. Enrichment Factor

An enrichment factor (*EF*) was applied to assess the contamination of SCSs in relation to reference elements, such as Al, Fe, Mn, Sc, or Ti [7]. *EF* is a significant indicator widely utilized for the quantitative evaluation of the degree of pollution and the discrimination of the source of metal pollution [41,42]. Matys Grygar and Popelka [43] proposed the use of Fe as a potential global reference element for the calculation of *EF* values. In this study, Fe was selected as the reference element. *EF* was defined as follows:

(1) $$EF=\frac{{\left(C_{i}/C_{\mathrm{Fe}}\right)}_{sample}}{{\left(C_{i}/C_{\mathrm{Fe}}\right)}_{Background}}$$

where ${\left(C_{i}/C_{\mathrm{Fe}}\right)}_{Sample}$ represents the ratio of element mass concentration ($C_{i}$) to Fe concentration ($C_{\mathrm{Fe}}$) in the sample and ${\left(C_{i}/C_{\mathrm{Fe}}\right)}_{Background}$ is the corresponding ratio in the background materials. LRB is located in southern China and the median values derived from statistical analysis of the elemental content SCSs of rivers in southern China by Cheng et al. [44] were selected as the background values for analysis. The levels classified are shown in Table S2.

#### 2.3.2. Potential Ecological Risk Index

The potential ecological risk index (RI) was originally introduced by Hakanson [45]. RI is widely employed to assess the potential ecological risk of elements in SCSs [16]. It was calculated as follows:

(2) $$RI=\sum_{i=1}^{n}E_{i}$$

where $E_{i}$ was the single potential ecological risk factor for element *I*, which was calculated as follows:

(3) $$E_{i}=f_{i}\times \frac{C_{i}}{B_{i}}$$

where $f_{i}$ is the toxicity coefficient of element *I*, as calculated by Hakanson [45] (e.g., Hg = 40, Cd = 30, As = 10, Pb = Cu = 5, Cr = 2, and Zn = 1), by Xu et al. [46] (e.g., Ni = Co = 5, Mn = Ti = 1), by Aksu et al. [47] (e.g., Sn = 4, Mo = 13), and by Wang et al. [48] (e.g., Be = 6, Sb = 5). $C_{i}$ is the analyzed value of the element *I* and $B_{i}$ is the background value of the element. The LRB is located in southern China and the median concentrations of SCSs from streams in southern China [44] can be appropriately used as background values. In this paper, the background values of P and the metals in SCSs from the LRB were adopted from Cheng et al. [44]. The classified risk levels are listed in Table S3.

#### 2.3.3. Statistical Analyses

Statistical analyses were conducted on the elements using SPSS software (version 25, IBM Corp., Armonk, NY, USA). Spearman correlation analysis (SCA) and principal component analysis (PCA), which interpret data by assessing the correlations between variables without spatial information, were applied to elucidate the relationships among the variables and assess the possible sources of pollution [49,50]. The data were converted to Z-scores prior to the analyses. Maps were drawn using OriginPro 2021 (version 9.8.0.200, OriginLab Corp., Northampton, MA, USA) and ArcGIS Desktop 10.7 (version 10.7.0.10450, Esri Inc., Redlands, CA, USA). All data input and basic statistical analyses, such as maximum, minimum, the arithmetic mean, median, standard deviation, and coefficient of variation of all primary data, were calculated by WPS Office.

## 3. Results

### 3.1. Concentrations of Pollutants

#### 3.1.1. Surface Water

The data and descriptive statistics of the dissolved and particulate-phase elements in the surface water samples from the LRB are presented in Tables S4, S5, S6, and S7. The data for the FW, SSs, and SCSs are also presented in Figures S1 and S2. To the best of our knowledge, this is the first study to systematically assess P and HMs pollution in SSs within the LRB. The results indicate that the higher element content values were located in the middle reaches of the main stream of the LR. These high values included elevated levels of dissolved As, Pb, Co, Zn, Mn, and P, as well as particulate-phase Co, Mn, Pb, As, Be, Cr, Cd, and P. When compared with the Chinese standards [51], the dissolved Cd, As, Cu, Pb, Zn, and Se concentrations at each site were below the Class I limits. The limits for these elements are listed in Table S5. In contrast, 100% of the samples were higher than the P limit of 0.02 mg/L stipulated by the Chinese standard. Approximately 25% and 33.3% of the sites exceeded the concentration limits of 0.1 mg/L and 5 μ/L for Mn and Sb, respectively. Collectively, these data indicate serious dissolved elemental pollution for Mn and Sb. In addition, the surface water from the tributaries of the LR also exhibited elevated element concentrations, particularly in Guiyu Town and Xiashan Town.

The rivers in Guiyu Town had higher element contents. Elevated values of dissolved Cu, Pb, Ni, Sb, Mo, and Sn were predominantly found in the upper and middle reaches of the Bergang River, while Cu, Pb, Ni, Co, Cr, Zn, Mn, and P were primarily present in the tributary (Guantian River) and the downstream part of the Bergang River. The HM concentrations in the SSs of the rivers in Guiyu Town were the highest among all of the sampling sites within the LRB (Figures S1 and S2), including the Sn, Sb, Cu, Ni, Pb, Be, Hg, and As contents in the upper and middle reaches of the Bergang River. In addition, the second highest Sn, Sb, Cu, and Pb contents occurred in the Old Lianjiang River. This indicates that the rivers in Guiyu Town were still polluted by wastewaters containing HMs after many years of regulated E-waste recycling activities (since 2012).

The Xiashan River had a significant effect on the HM contents of the mainstream of the LR. The contents of dissolved Cu, Ni, Zn, Cd, and Se were significantly elevated. Zn, Cd, and Se reached their peak levels, and the particulate-phase Cd, Mo, Se, Cr, and Zn reached their highest values. Unlike the irregular trends of the dissolved elements, the high contents of the metals occurred in the mid-mainstream of the LR (e.g., S9, S10, and S11). With the exception of Cr, the order of the concentrations was as follows: S9 < S10 < S11. For Cr, the order was S10 < S9 < S11. These trends indicate a high level of spatial consistency, suggesting that the surface water in the downstream section of the LR (S11) was influenced by pollution originating from upstream point sources (e.g., the Xiashan River).

#### 3.1.2. Surficial Channel Sediments

The data and descriptive statistics for the pH, OM, and sediment-associated HMs analyzed in 23 SCS samples from the studied basin are presented in Tables S8 and S9. Chinese standards (Table S10) [52] were utilized to assess the degrees of HM contamination in the SCSs. The concentrations of Cu, Zn, Hg, Pb, Cd, and Cr in SCSs from the LRB exceeded Grade I at 73.9%, 69.6%, 65.2%, 60.9%, 34.8%, and 26.1% of the sites, respectively. A total of 26.1%, 8.7%, and 4.3% of the sites exceeded Grade III concentrations for Cu, Hg, and Zn, respectively.

Spatially, the highest values of Cu and Sb were detected in tributaries (e.g., the Bergang River and Xiashan River) or within the upstream areas of the LRB. Nickel, for example, is enriched in SCSs of Guiyu Town [19,23,25,27]. Chromium (Cr) and Ni were uniformly distributed along the LRB, with enrichment in the Bergang River, Xiashan River, and Qiufeng River, as well as along the mainstream of the LR. Higher levels of pollutants were identified at S1 (rather than at S2), indicating that the metals were primarily derived from a point source on the north side of the LRB, such as the Old Lianjiang River, which is renowned for its long-standing E-waste recycling activities.

### 3.2. Spearman Correlation Analysis

SCA among pollutants was used to elucidate the inter-relationships between various pollutants present in the FW, SSs, and SCSs, thereby providing insights into their potential source similarities and behavior in the environment [53].

#### 3.2.1. Dissolved Constituents

During the SCA of the dissolved elements, Hg and Be were not included in the SCA analysis because their concentrations were below the detection limits. The SCA results are presented in Figure S3. A number of elements exhibited significant positive correlations (*p* < 0.05). Specifically, Cd exhibited positive correlations with Se and Cu; Cu exhibited a positive correlation with Ni; Sb exhibited positive correlations with Sn and Mo; Mn exhibited positive correlations with Zn, P, Co, and Pb; and As exhibited positive correlations with Co, Mo, P, and Ti. The dissolved oxygen (DO) and pH levels did not exhibit statistically significant correlations with the concentrations of the dissolved metals.

Dissolved metals are regarded as the most mobile, active, and bioavailable components in aquatic systems [54], suggesting the existence of immediate or recent pollutant inputs [55]. Wong et al. [54] reported that the concentrations of dissolved Cu, Ni, Sb, Mo, Pb, and Cd were significantly higher in the Bergang River than in the Xieyao reservoir in Guiyu Town, and they suggested that these HMs were sourced to local E-waste recycling activities. The elevated concentrations of Cu, Ni, Sb, Mo, and Sn in the Bergang River may be due to the E-waste recycling and other human activities along the Bergang River, despite years of the sustained implementation of pollution control policies and activities.

Specifically, the higher levels of dissolved Pb, Co, Zn, Mn, and P in the Guantian River and the middle reaches of the main stream of the LR, the higher levels of dissolved Cd and Se in the Xiashan River, and the higher levels of dissolved As, Co, Mo, and P in the estuary of the Zhonggang River were attributed to various anthropogenic activities, including industrial discharges and domestic sewage. These findings highlight the heterogeneous nature of the pollution across different rivers within the LRB.

#### 3.2.2. Suspended Solids

The results of the SCA (Figure S4) indicate that Ti, Fe, and Al were strongly correlated with one another (*p* < 0.01) and significantly correlated with Hg, Co, Cr, and Cu. In addition, Ti, Be, and As were also strongly correlated and significantly correlated with Co, Cr, Pb, Sb, Sn, and Cu (except for As-Co, As-Cr, and Sn-Ti). Generally, concentrations of Ti, Fe, and Al are attributed to a natural source [56,57], suggesting that natural sources were an important source of HMs in the SSs of the LRB.

Zinc, Se, Mn, Co, and P were significantly correlated (*p* < 0.05, except for Se-Co) and were significantly correlated with Cu, Ni, Cd, and Mo (*p* < 0.05, except for Mn-Cu, P-Cu, and P-Ni). Manganese, Co, and P were mainly detected in the Bergang, Xiashan, and Chendian rivers and the middle reaches of the main stream of the LR, and they had C.V. values ranging from 63% to 109%, suggesting an anthropogenic source (s).

Molybdenum, Cd, Ni, Cu, Sn, Sb, Pb, Cr, Zn, and Se were significantly positively correlated (*p* < 0.05, except for Cr-Sn, Cr-Sb, and Cr-Se). Similar to the FW, which had higher amounts of dissolved Cu, Ni, Sb, Mo, Sn, Pb, Cd, and Ni mainly in the Bergang and Xiashan rivers, the highest concentrations of most of these HMs were observed along the Bergang River, Xiashan River, Old Lianjiang River, and Jinxi River. This indicates that the metals in the FW and SSs of these rivers likely originated from similar sources.

#### 3.2.3. Surficial Channel Sediments

SCA was carried out on the pH, OM, and the analyzed heavy elements in the SCSs of the LRB to assess the consistency of the sources of the pollutants. Most of the previous literature suggests that the solubility, mobility, adsorption, and precipitation of HMs are influenced by the pH [13,58]. However, negative correlations were generally observed between the pH and OM. No statistically significant correlations were observed between pH and the other elements (Figure S5), suggesting that pH is not a primary control of pollutant concentrations within the SCSs in the LRB.

In the LRB, conservative elements that are highly stable in the crust (Al, Fe, Mn, and Ti) [56,57] are significantly and positively correlated with each other (*p* < 0.05, except for Al-Fe, Al-Mn, Figure S5). Aluminum, Fe, Mn, and Ti were also significantly correlated with Be, Co, and Sb (except for Mn-Be, Ti-Sb). Aluminum, Ti, Fe, and Co are considered conservative elements and rarely undergo migration in most environments [16]. These metals were probably derived from the parent rocks and underwent secondary enrichment during weathering [15]. In addition, Be, Co, Mn, Fe, Ti, and Al in the current study exhibited a low C.V. (<29%, Table S9) and a more even spatial distribution, indicating that they were less influenced by differences in human activities [16].

The other metals, along with OM, were uncorrelated with Be, Ti, Mn, Fe, and Al, a finding consistent with the work of Shi [28]. A weak correlation of Cd, Cu, Ni, Pb, Zn, and Hg with Al and Fe in SCSs was found (*R*^2^ ranges from 0.12 to 0.54), indicating that the source contribution of metals was dominated by human activities. SCSs contain substances, such as clay, humic acid, and OM, which complex with metals [42]. Metal-OM and metal–fine grain complexes are important mechanisms for the deposition of metals in the aquatic systems [59], while the influence of the latter appeared to be relatively minor in the LRB.

OM, Zn, Mo, Hg, Sn, P, Pb, Se, Cr, Cd, Sb, Cu, and Ni exhibited significant positive correlations (except for Pb-Cr and Ni-Se). Arsenic was positively correlated with Zn, Mo, Pb, Se, Cd, Sb, Cu, and Be. Beryllium was also correlated with Co, As, Sb, and Cu (Figure S5). The majority of these contaminants had C.V. values higher than 45% (except for Be and Co, Table S9), indicating that OM, P, and HMs in the SCSs of the LR predominantly originated from human activities.

### 3.3. Enrichment Factors

*EF*s were calculated to determine the extent of elemental contamination in SSs and SCSs (Figure S6, Table S11). There were average *EF*s of As, Cr, and Al in the SSs, and of Al, Cr, Co, As, and Mn in SCSs < 1.5 (Table S11), indicating that the SSs and SCSs in the LRB were generally not contaminated by these metals [60]. The average *EF*s of Sn fell within the very high enrichment category for both the SSs and SCSs. The values of Sb, Mn, P, Hg, Zn, Se, and Cd in the SSs, and Sb, Cu, Hg, and Cd in the SCSs reached significant enrichment. The values of Cu, Pb, Ni, and Mo in the SSs, and of Zn, Pb, Mo, Be, and Se in the SCSs fell within the moderate enrichment levels, whereas those for other elements had minimal enrichment.

In the SCSs, a few elements exhibited significant-to-extremely high enrichment, including Sn, Sb, Cu, Hg, and Cd, mainly along the Bergang, Xiashan, and Hucheng rivers and the mainstream of the LR. In the SSs, several elements exhibited significant-to-extremely high enrichment, including Sn, Sb, Mn, and P, which were found in most sites within the LRB. Additionally, Hg, Cd, and Cu were mainly enriched in the Bergang River, Old Lianjiang River, Xiashan River, and the mainstream of the LR. Zinc and Se exhibited similar enrichment patterns, primarily in the Chendian River, Old Lianjiang River, Xiashan River, and the mainstream of the LR. 

For Sn, Sb, Cu, Hg, and Cd, 91.3%, 47.8%, 47.8%, 47.8%, and 34.8% of the *EF*s of all of the SCS samples were higher than five, respectively. In the SSs, the percentages of the samples with *EF*s greater than five for Sn, Sb, P, Mn, Hg, Cu, Cd, Zn, and Se were 79.2%, 70.8%, 58.3%, 54.2%, 41.7%, 41.7%, 37.5%, 37.5%, and 25.0%, respectively. As the primary scavenger of pollutants in river water, SSs are often enriched with numerous pollutants (e.g., HMs) [61]. The *EF*s for Sn, Sb, Cu, Mn, P, Cd, Se, Zn, Co, Pb, Ni, and Mo at most of the sites were higher in the SSs than those found in SCSs, especially along the Bergang River, Chendian River, Old Lianjiang River, and Guitou river.

### 3.4. Pollutant Source Identification

**Figure 2:**
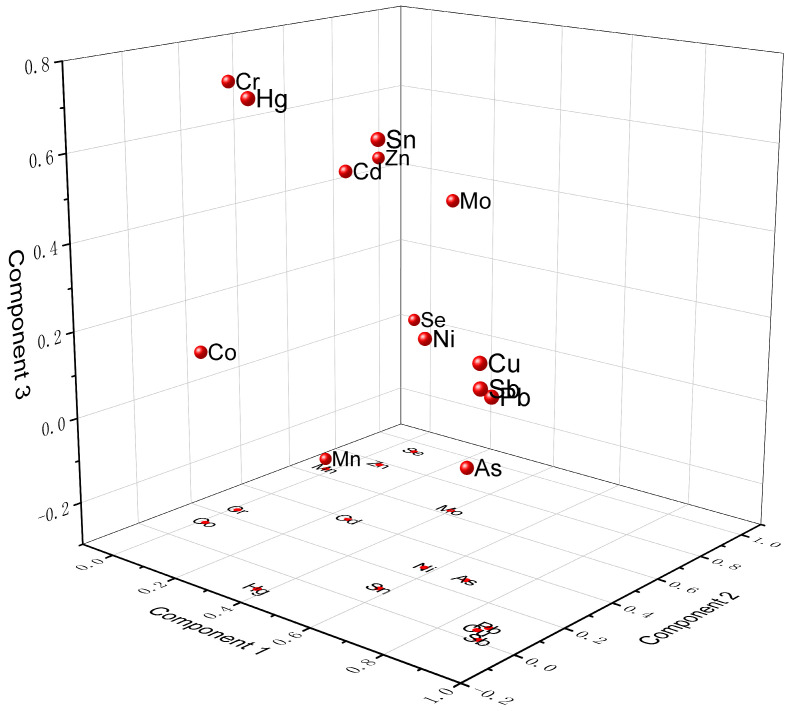
Plot of the PCA component loadings for the suspended solids (SSs) and surficial channel sediments (SCSs).

**Figure 3:**
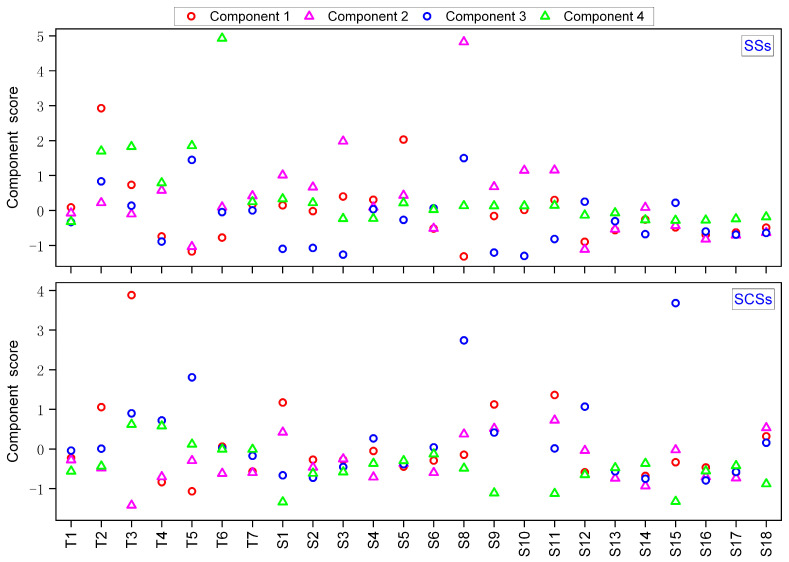
Variations in the component scores derived from the PCA of the suspended solids (SSs) and surficial channel sediments (SCSs).

The dissolved and sediment-associated pollutants in surface water can be derived from natural and/or anthropogenic sources. SCSs contamination represents historically polluted deposits, and the result may be influenced by human disturbances, such as dredging and engineering construction. To further evaluate the anthropogenic sources of the pollutants in the SSs and SCSs in the LRB, PCA was conducted. The data for the anthropogenic metals were standardized prior to analysis to account for differences in the units of the parameters. The Kaiser–Meyer–Olkin (KMO) test and Bartlett sphericity test were implemented on the pollutants to determine the applicability of the data to the PCA [62]. In this study, the KMO value of the 14 metals was 0.685 and significance was defined as the 99% probability level (*p* < 0.001). Four components were extracted and the cumulative variance contribution rate was 78.398% (Table S12, Figure 2 and Figure 3).

#### 3.4.1. E-Waste Recycling Activities

For the SSs and SCSs, the first component (PC 1) accounted for 27.455% of the total variance and had the highest loadings for Sb, Pb, Cu, As, Sn, and Ni. These loadings are similar to the results of the SCA and indicate that the elements have a similar spatial distribution [63]. Our results are inconsistent with the PCA results of Mao et al. [21], who suggest that Cu, Ni, Pb, Mn, and Zn represent a specific pollution pattern within Guiyu Town, with Zn and Sn representing local industrial and manufacturing sources. The difference in PCA results might be associated with different sampling times (>7 years) and sites.

In this study, the highest Sb, Sn, Pb, Cu, Ni, and As concentrations in the FW, SSs, and SCSs were mostly observed in the middle and upper reaches of the Bergang River. Additionally, these HMs were also found in the SSs in the Old Lianjiang River, as well as in the SCSs in the middle and lower reaches of the main stream of the LR. Wong et al. [22] reported that the concentrations of dissolved Ni, Pb, Sb, As, Cu, and Cd in the Bergang River and the Old Lianjiang River were 4–315 times higher than those in the Xieyao Reservoir in Guiyu Town. These pollutants are attributed to contamination originating from local E-waste recycling activities [22]. Mao et al. [21] reported that the maximum Sb, Pb, Cu, Sn, and Ni concentrations occurred in SCSs from Guiyu Town, and their concentrations were several times-to-two orders of magnitude higher than those in the upper and lower reaches of the LRB. Other reports indicate that the high pollution of Sn and Sb [24,25], Cu and Pb [19,20,22,23,24,25,27], Ni [19,20,23,25,27], and As [25] occurred in SCSs from rivers of the Guiyu Town. This indicates that the HMs (e.g., Sb, Pb, Cu, As, Sn, and Ni) in the FW, SSs, and SCSs were likely derived from human activities along the Bergang River and Old Lianjiang River, such as E-waste recycling activities.

#### 3.4.2. Mixed Pollution from Human Activities

For the SSs and SCSs, the second component (PC 2) accounted for 19.325% of the total variance. It was heavily loaded by Se, Zn, Mn, and Mo, analogous to the results of the SCA. In the FW, the highest dissolved Mn contents were mainly detected in rivers in Gurao Town and the middle of the mainstream of the LR, while the highest Zn and Se contents were detected in the Xiashan River. The highest levels of Se, Zn, Mn, and Mo in the SSs and SCSs were predominantly detected in the Xiashan River and the mainstream of the LR. Higher contents of these elements were also detected in the SSs in the Chendian River, the lower part of the mainstream of the Bergang River and its tributary, and the Old Lianjiang River. These areas are associated with industrial activity and possess relatively high population densities.

Zhu et al. [18] concluded that Zn and Mo were derived from industrial activities, and Zn was identified in urban domestic sewage [64]. Additionally, Mo is commonly employed in welding materials [65]. Nonetheless, During the past few decades, the environmental protection, awareness, and legal consciousness of practitioners has remained inadequate [66], resulting in the disposal of large volumes of mixed waste to aquatic systems, including sewage effluent and domestic wastewater, industrial effluents, and agricultural sewage. This gives rise to the challenge of identifying the precise sources of pollutants. The elements mentioned above may be associated with mixed pollution sources from human activities, such as industrial, domestic, and agricultural pollution. Their associations with Mn suggest that the migration of Se, Zn, and Mo occurs as constituents sorbed onto Mn oxides and hydroxides. Component 2 suggests that there were multiple sources of these elements, including a mixed array of sources from anthropogenic activities.

#### 3.4.3. Metal Processing

Component 3 (PC 3) of the SSs and SCSs is primarily loaded by Hg, Cr, Sn, and Cd, accounting for 18.541% of the total variance. For the SSs and SCSs, the highest Hg, Cr, Sn, and Cd contents were mainly detected in the Xiashan River, the tributary of the Bergang River (Guantian River), the Hucheng River, the Zhonggang River, and the Jinxi River. Higher Hg, Cr, Sn, and Cd contents were also detected in the SSs in the middle and upper reaches of the Bergang River and in the SCSs in the middle of the mainstream of the Bergang River and its tributary (Liyubo River). This pattern was similar to that of the dissolved Cd in the Xiashan River and the upper reaches of the Bergang River. Previous studies have found that the primary sources of Cd included the electroplating industry [64,67] and other metal processing activities [68]. Therefore, it can be hypothesized that the sources associated with PC 3 are likely metal-processing activities.

#### 3.4.4. Battery Manufacture and Recycling

For the SSs and SCSs, Component 4 (PC 4) accounts for 13.076% of the total variance, and it is primarily loaded by Co, Ni, and Mn. Higher concentrations of particulate-phase Co and Ni were detected in the Bergang River (except for the upper section), which is similar to the higher dissolved Co and Mn contents in the Bergang River and the middle of the mainstream of the LR, as well as the SCSs in the middle of the mainstream of the Bergang River and its tributary (Liyubo River). In addition, the sites with the six highest Fe contents were located within the Bergang River. Nickle is associated with battery production [69]. Cobalt is utilized in the production of rechargeable batteries for both laptops and mobile phones [70], and Fe-Mn oxides are often found to be principal factors influencing Co accumulation as it possesses an extremely strong specific adsorption for Fe-Mn oxides/hydroxides [71]. These relations suggested that pollutants associated with PC 4 were likely affected by the manufacture and recycling of batteries containing Co. Moreover, the Co and Ni in the SSs in the Bergang River were primarily influenced by Fe-Mn oxides/hydroxides.

### 3.5. Risk Assessment of Metals

**Figure 4:**
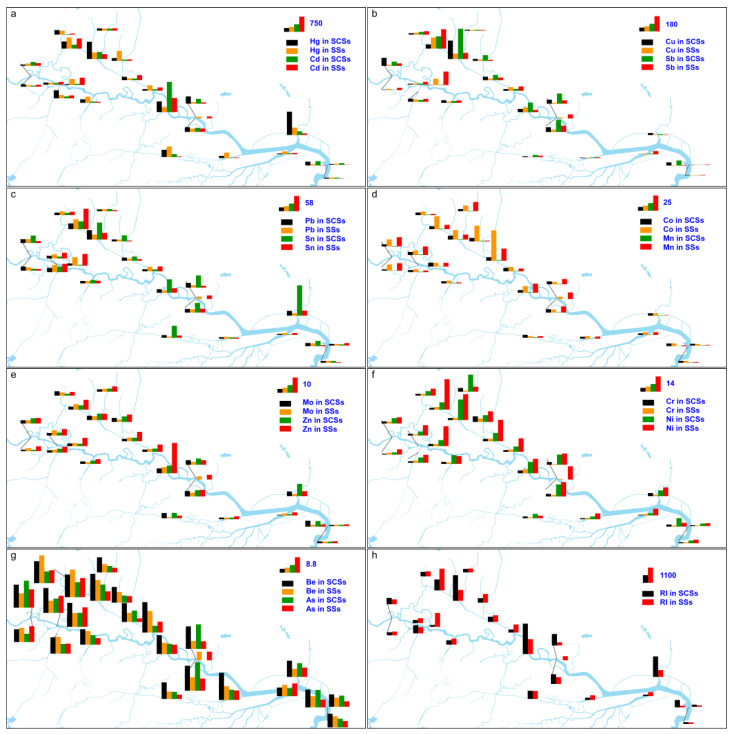
Potential ecological risk for metals in suspended solids (SSs) and surficial channel sediments (SCSs) from the LRB. (**a**) Hg and Cd; (**b**) Cu and Sb; (**c**) Pb and Sn; (**d**) Co and Mn; (**e**) Mo and Zn; (**f**) Cr and Ni; (**g**) Be and As; (**h**) potential ecological risk index (RI).

The potential ecological risk was evaluated through the analysis of a risk index (RI). The statistical results of the potential ecological risk associated with SSs and SCSs for each sampling site are presented in Figure 4. It is important to note that the metal concentrations utilized for the risk assessment conducted in this study include both the bioavailable and non-bioavailable fractions. In the SS and SCS samples, the values for individual metals at each sampling site are separately listed in Tables S13 and S14, respectively. In the case of the SSs, the proportion of sites with $E_{i}$ ≥40 for Hg, Cd, Sn, Sb, Cu, Mo, and Co were 100%, 80%, 52%, 40%, 20%, 12%, and 4%, respectively. In the case of the SCSs, 95.5%, 91.3%, 56.5%, 39.1%, 26.1%, and 26.1% of the sites exhibited $E_{i}$ ≥40 for Hg, Cd, Sn, Sb, Cu, and Mo, respectively. For the remaining HMs, all sites exhibited low-risk levels.

#### 3.5.1. Surficial Channel Sediments

In the SCSs from the LRB, the maximum values of Hg, Cd, Sn, Sb, Cu, and Mo were 1146, 1492, 463, 361, 220, and 52, respectively. Mao et al. [21] report that Sb in most SCSs from the LRB exhibited very high ecological risk levels, whereas Cu, Ni, and Pb possessed higher risk levels at some sites. Nickle, Zn, As, and Pb predominantly exhibited moderate-to-low levels of ecological risk, whereas Cr, Mn, Co, Cd, and Hg are typically categorized as low-risk elements [21]. The results derived from this study exhibit significant deviations when compared to the findings by Mao et al. [21], especially the primary risk contributors. For Hg, Cd, Sn, Sb, and Cu in some of the rivers, the values reached very high and high levels, namely, sediments from the Bergang River, Xiashan River, Zhonggang River, Hucheng River, and mainstream of the LR.

Approximately seven years ago, as reported by Mao et al. [21], the RI values for 77.4% of the samples were very high, with 19.4% falling within the moderate or comparatively high-risk category. During the present study, the decreasing order of RI values by sites (Table S14) was S8 > T3 > S15 > S9 > T2 > S11 > S12 > S4 > S18 > S1 > T6 > T5 > T1 > S6 > T4 > S3 > T7. The ten sites with the highest RI values were classified as being very high, while the subsequent seven sites were categorized as having a considerable risk. In total, these sites accounted for 73.9% of the sites. Five sites also possessed a moderate risk and the remaining site was classified as having a low risk. Evidently, following years of implemented governance to remediate the pollution associated with E-waste recycling activities in Guiyu Town, and the comprehensive management of the LRB from 2018 to 2020, the overall potential ecological risks in SCSs have declined. Nonetheless, despite the implementation of comprehensive treatment measures, the SCSs within the LRB continue to pose significant potential risks to the aquatic ecosystem. Notably, risks were more pronounced within the Bergang River, Hucheng River, Xiashan River, Jinxi River, and Zhonggang River, as well as the mainstream of the LR.

#### 3.5.2. Suspended Solids

Among the SS samples, Hg, Cd, Sn, Sb, and Cu concentrations corresponding to moderate- or higher-risk levels were detected in the Bergang River and Old Lianjiang River; Hg, Cd, Sn, and Cu concentrations corresponding to moderate- or higher-risk levels were detected in the Xiashan River; Hg, Cd, and Sn concentrations corresponding to moderate- or higher-risk levels were detected in the other tributaries of the LR; and Hg, Cd, Sn, and Sb concentrations corresponding to moderate- or higher-risk levels were detected in the lower reaches of the mainstream of the LR. In this study, we determined that the order of the RI values of the sites (Table S13) was as follows: T2 > S8 > S5 > T7 > T3 > T5 > S12 > S15 > S11 > S4 > S6 > T6 > S3 > T1 > S1 > S13 > S10 > S14. The seven sites with the highest RI values were classified as being very high risk, while the other sites were categorized as having a considerable risk.

The SSs in the surface water of several tributaries of the LR continue to exhibit high- or very-high-risk levels of HMs, suggesting ongoing HMs pollution in the Bergang River, Xiashan River, Old Lianjiang River, Zhonggang River, and Hucheng River. For instance, characteristic pollutants linked to E-waste recycling were identified in rivers within Guiyu Town (e.g., the Bergang River and Old Lianjiang River). These contaminants might pose varying degrees of ecological risk to the mainstream of the LR, its estuary, and the South China Sea.

## 4. Discussion

### 4.1. Governance Outcomes

Since 2012, Guiyu Town has implemented stringent measures to regulate E-waste sources and rigorously enforced actions against illegal E-waste recycling activities, such as dismantling acidic leaching and incineration facilities [66]. The Circular Economy Industrial Park (CEIP) was established and became operational by the end of 2015, effectively consolidating scattered polluting activities within a designated area, thereby facilitating the industrialization, scalability, and sustainable development of E-waste recycling. Additionally, remediation (e.g., the dredging and reinforcement of river embankments) has been conducted in the Bergang River since October 2017. The river environment management in Guiyu Town, as mentioned above, contributed to the reduction of the pollutant levels, the enhancement of the photosynthetic organism abundance in the surface water, and, consequently, the increase in the dissolved oxygen content [66].

In the LRB, the long-term insufficient replenishment of fresh water, coupled with measures taken to prevent seawater intrusion, has resulted in the construction of sluice gates on many tributaries of the LR before their confluence with the main river channel. These structures have been maintained in a closed state for extended periods to facilitate water storage for agricultural irrigation and other uses (except for flood discharge purposes). Consequently, the hydrological regime of the main stream of the LR and its tributaries, such as the Bergang River and the Old Lianjiang River, has been significantly altered, characterized by markedly reduced flow velocities. In this study, the pH and dissolved oxygen values of the surface water in the rivers in Guiyu Town were determined to be 6.9–8.7 and 4.2–9.4 mg/L, respectively. The dissolved metal contents in these rivers were significantly lower than those measured by Wong et al. [54], except for Mo, Cr, and Pb (Figure S7). In this study, we found that the pH values were 5.79–6.88 and the rates of the decrease in the metal contents were greater than 40% (Figure S7).

Notable discrepancies in the pollutant levels have been documented in SCSs from the Bergang River and the mainstream of the LR in multiple previous studies (Tables S15 and S16; Figure S8). Compared with the results of Wu et al. [25] for July 2014, the Sb and Sn concentrations in the Bergang River decreased by more than 80%, while the Cu, Pb, and Cd concentrations decreased by more than 39%. Compared with the findings of Mao et al. [21] for January 2013, which covered rivers within Guiyu Town and the Guiyu section of the LRB, the Sb, Sn, Ni, Cu, Zn, Pb, Mo, and Cd concentrations in the Bergang River decreased by over 80%. In the LRB (excluding the Bergang and Old Lianjiang rivers), compared with the concentrations in the downstream part of the LRB reported by Mao et al. [21], the Sb, Sn, Cr, Ni, Cu, Zn, Mo, and Cd concentrations decreased by more than 30%. As indicated by the aforementioned data, the environmental governance measures implemented in the LRB has significantly improved the sediment environmental quality in the LRB. However, certain governance gaps persist, particularly in the terminal tributary regions of the Bergang River and Old Lianjiang River. Compared to those reported by Xiong et al. [27] for April 2021, the mean Cu, Cd, Zn, and Pb contents in the Bergang River were lower.

### 4.2. The Role of OM in Element Deposition

OM plays an important role in pollutant adsorption and precipitation [59]. In this study, the amount of OM was significantly positively correlated with P and HMs. OM may sorb HMs and, therefore, play an important role in the deposition of HMs within the SCSs of the LRB. The generated SCA data show that SCSs across all sites in the LRB were statistically correlated (except for Co, Be, Ti, Mn, Fe, and Al), implying they likely originated from a common source (s) and/or behaved similarly in the aquatic environment. These relations suggest that SCS-associated metals in the LRB were jointly influenced by OM, Mn oxyhydroxides, and the sediment grain size. Meanwhile, in the midstream and estuary of the LR, water conservancy infrastructure—specifically sluices—has been implemented to retain freshwater and mitigate the effects of seawater intrusion. The establishment of metal-OM and metal-SS complexes may be promoted by the poor fluidity of the water body in the LRB due to the long-term impounding of the LRB estuary and little freshwater replenishment.

### 4.3. Integrated Management Strategies for the River Basins

Based on the findings of this study, targeted water governance strategies can be effectively implemented to address specific environmental challenges in aquatic systems. According to the research findings of Du et al. [26] and Xiong et al. [27], elevated concentrations of HMs continued to be present in SCSs in the uppermost tributaries of the Old Lianjiang River and the Bergang River in Guiyu Town. In this study, we found that the values of the dissolved and SS/SCS-associated HMs in the Bergang River and Old Lianjiang River were significantly higher than those in other rivers, including Sb, Pb, Cu, As, Sn, and Ni. These elevated levels of HMs exhibited higher enrichment and distinct characteristics associated with E-waste recycling activities, suggesting that the local environment remains impacted by contaminants from wastewater and solid waste generated during E-waste recycling activities.

Moreover, certain tributaries may still contain sediments with high amounts of HMs that have not been adequately removed. The resuspension or disturbance of such sediments could negatively affect the quality of the downstream surface water and sediments. The high content, enrichment, and ecological risks of HMs are consistent with pollution patterns from the E-waste recycling industry of downstream surface water and sediments and show the enhancement of the dredging efforts. Therefore, it is recommended that dredging operations be conducted in a hierarchical and sequential manner, starting from the terminal sections of tributaries, followed by the tributaries themselves, and finally, the main stream of the Bergang River. This approach is designed to prevent the recontamination of the Bergang River by high-concentration HMs. This sequential dredging strategy has a broad applicability and could be effectively implemented in other contaminated aquatic systems.

According to the research findings of Li et al. [20] and Du et al. [26], elevated concentrations of HMs continued to be present in SCSs in the mainstream of the LR. In the present study, other rivers in the LRB also exhibited elevated levels of dissolved and SS/SCS-associated HMs, particularly the Xiashan River (e.g., Cu, Ni, Zn, Cd, and Se), the tributary of the Bergang River (the Guantian River, e.g., Co, Cr, and Zn), and the upper reaches of the mainstream of the LR (e.g., Cu, Cd, As, Mn, and P). Furthermore, sediments across multiple river systems exhibited significant HM contamination and posed high-to-very high ecological risks, with particularly severe risk levels in the Xiashan River, Hucheng River, Jinxi River, Zhonggang River, and the mainstream of the LR. Significant inter-river variations in the compositions of the high-value HMs were evident.

The potential pollution sources were primarily attributed to industrial discharges, domestic wastewater, and other anthropogenic activities, underscoring the importance of source identification in the formulation and implementation of effective remediation measures. Therefore, it is recommended that remediation strategies similar to those implemented in Guiyu Town be adopted in these rivers to prevent recontamination and ensure long-term environmental safety.

## 5. Conclusions

Samples of dissolved and suspended solids are prone to significant short-term variations associated with changes in the flow conditions, while the analysis of surficial channel sediments provides average element abundances for periods ranging from a few weeks to years. Thus, sediments provide a longer-term view of the changes that may occur in response to implemented remediation strategies. However, despite several years of environmental remediation, the contamination reported in this study indicates that HMs still pose a significant potential ecological risk.

The presence of dissolved and particulate anthropogenic pollutants in the surface water samples suggests the potential occurrence of point-source pollution or the influence of upstream anthropogenic activities, such as sediment dredging, the construction of sluice gates, and the stabilization of river embankments, which may cause sediment resuspension and affect the quality of the surface water. It is imperative to strengthen the implementation of pollution source control, sewage interception, and dredging operations in terminal tributaries.

Sediment, as a potential secondary pollution source, presents considerable ecological risks. Remediation strategies should be systematically implemented, progressing from terminal tributaries to secondary tributaries to primary tributaries and, ultimately, to the mainstream of the LR. Sediments generated during dredging should be handled and disposed of by accredited professional agencies. The aforementioned recommendations may also be applicable to the remediation of other contaminated rivers.

## Data Availability

The data presented in this study are available from the corresponding author upon reasonable request.

## Supplementary Materials

The following supporting information can be downloaded at: https://www.mdpi.com/article/10.3390/toxics13100840/s1. Text S1: Sampling collection and determination. Table S1: Physical characterization of surface water samples; Table S2: Enrichment levels by Sutherland [72] and Loska et al. [73]; Table S3: Risk levels by Håkanson [45]; Table S4: Results of dissolved element contents in surface water; Table S5: Statistical results of dissolved elemental contents in surface water; Table S6: Results of particulate-phase elemental contents in surface water; Table S7: Statistical results of particulate-phase elemental content in surface water; Table S8: Results of pH, organic matter (OM), phosphorus (P), and metal contents in surficial channel sediments; Table S9: Statistical results of pH, organic matter (OM), phosphorus (P), and metal contents in surficial channel sediments; Table S10: Standards for marine sediment quality [52]; Table S11: Enrichment factors of elements detected in suspended solids (SSs) and surficial channel sediments (SCSs); Table S12: Analysis of the main components of pollutants in suspended solids in surface water and surficial channel sediments; Table S13: The potential ecological risk assessment for suspended solids in surface water; Table S14: The potential ecological risk assessment for surficial channel sediments in the Lianjiang River Basin; Table S15: Comparison of pollutant concentrations in surficial channel sediments in rivers in Guiyu Town, China; Table S16: Comparison of pollutant concentrations in surficial channel sediments in Lianjiang River Basin, and data on pollutant concentrations from other studies; Figure S1: The distribution profiles of of dissolved element concentrations in surface water (FW), dissolved elements in suspended solids (SSs), and pollutants in surficial channel sediments (SCSs); Figure S2: The distribution profiles of of dissolved element concentrations in surface water (FW), dissolved elements in suspended solids (SSs), and pollutants in surficial channel sediments (SCSs); Figure S3: Spearman correlation coefficient matrix for dissolved elemental concentrations, pH values, and dissolved oxygen (DO) contents in surface waters; Figure S4: Spearman correlation coefficient matrix for suspended solids-associated elemental concentrations in surface waters; Figure S5: Spearman correlation coefficient matrix for pollutants in surficial channel sediments; Figure S6: Enrichment factors of elements detected in suspended solids (SSs) and surficial channel sediments (SCSs); Figure S7: Comparison of dissolved elemental values in surface water in Guiyu Town; Figure S8: Comparison of metal values in surficial channel sediments in the Lianjiang River Basin. (References [13,15,16,18,19,20,21,22,23,24,25,26,27,28,30,31,32,33,34,35,36,37,38,39,40,44,45,46,47,48,51,52,54,72,73,74,75,76,77] are cited in the Supplementary Materials).

## Abbreviations

The following abbreviations are used in this manuscript:

AFS	Atomic Fluorescence Spectrometry
CEIP	Circular Economy Industrial Park
CND	Chaonan District
CYD	Chaoyang District
DO	dissolved oxygen
EF	enrichment factor
EFs	enrichment factor values
E-waste	electronic waste
FW	filtered water
HM	heavy metal
HMs	heavy metals
ICP-MS	inductively coupled plasma mass spectrometry
ICP-OES	inductively coupled plasma optical emission spectrometry
LR	Lianjiang River
LRB	Lianjiang River Basin
PCA	principal component analysis
RI	risk index
SCA	Spearman correlation analysis
SCSs	surficial channel sediments
SSs	suspended solids
