# Supplementary material for: Pollution Status, Ecological Risks, and Potential Sources of Metals in the Middle and Lower Reaches of the Lianjiang River Basin, Guangdong Province, China
Source: Toxics. 2025 Oct 1;13(10):840. doi: 10.3390/toxics13100840 (PMC12567751; doi:10.3390/toxics13100840)
Supplement: Supplementary file 1 [file toxics-13-00840-s001.zip › toxics-3875072-supplementary.pdf]

Supplementary information

for

Pollution Status, Ecological Risks, and Potential Sources of Metals in the Middle and Lower Reaches of the Lianjiang River Basin, Guangdong Province, China

Yongzhong Lai [1,2,*], Le Li [2] , Xianbing Huang [2], Guoyong Lu [2], Fengqin Pan [2], and Wenhua Liu [1]

[1] Provincial Key Laboratory of Marine Biotechnology, Institute of Marine Science, Shantou University, Shantou 515063, China; whliu@stu.edu.cn

[2] Guangdong Shantou Ecological, and Environment Monitoring Center Station, Shantou 515041, China; 13790848334@163.com (L.L.); 13414084791@163.com (X.H.); stlugy@163.com (G.L.); 15019708363@163.com (F.P.)

* Correspondence: 17yzlai@stu.edu.cn

Index for Supporting Information

Text S1 *Sampling collection and determination* 1

Table S1 *Physical characterization of surface water samples* 3

Table S2 *Enrichment levels by Sutherland[72] and Loska et al. [73]* 4

Table S3 *Risk levels by Håkanson[45]* 4

Table S4 *Results of dissolved element contents in surface water* 5

Table S5 *Statistical results of dissolved elemental contents in surface water* 6

Table S6 *Results of particulate-phase elemental contents in surface water* 7

Table S7 *Statistical results of particulate-phase elemental content in surface water* 8

Table S8 *Results of pH, organic matter (OM), phosphorus (P), and metal contents in surficial channel sediments* 9

Table S9 *Statistical results of pH, organic matter (OM), phosphorus (P), and metal contents in surficial channel sediments* 10

Table S10 *Standards for marine sediment quality [52]* 11

Table S11 *Enrichment factors of elements detected in suspended solids (SSs) and surficial channel sediments (SCSs)* 12

Table S12 *Analysis of the main components of pollutants in suspended solids in surface water and surficial channel sediments* 13

Table S13 *The potential ecological risk assessment for suspended solids in surface water* 14

Table S14 *The potential ecological risk assessment for surficial channel sediments in the Lianjiang River Basin* 15

Table S15 *Comparison of pollutant concentrations in surficial channel sediments in rivers in Guiyu Town, China* 16

Table S16 *Comparison of pollutant concentrations in surficial channel sediments in Lianjiang River Basin, and data on pollutant concentrations from other studies* 17

Figure S1 *The distribution profiles of of dissolved element concentrations in surface water (FW), dissolved elements in suspended solids (SSs), and pollutants in surficial channel sediments (SCSs)* 18

Figure S2 *The distribution profiles of of dissolved element concentrations in surface water (FW), dissolved elements in suspended solids (SSs), and pollutants in surficial channel sediments (SCSs)* 19

Figure S3 *Spearman correlation coefficient matrix for dissolved elemental concentrations, pH values, and dissolved oxygen (DO) contents in surface waters* 20

Figure S4 *Spearman correlation coefficient matrix for suspended solids-associated elemental concentrations in surface waters* 21

Figure S5 *Spearman correlation coefficient matrix for pollutants in surficial channel sediments* 22

Figure S6 *Enrichment factors of elements detected in suspended solids (SSs) and surficial channel sediments (SCSs)* 23

Figure S7 *Comparison of dissolved elemental values in surface water in Guiyu Town* 24

Figure S8 *Comparison of metal values in surficial channel sediments in the Lianjiang River Basin* 25

Text S1 *Sampling collection and determination*

A total of 24 sampling sites (presented in Figure 1), encompassing the mainstream, and main tributaries, were selected. These included 7 sites in Bergang River (BGR; T1–T7), 1–2 sites in main tributaries such as Chendian River (CDR; S3), Jinxi River (JXR; S4), Old Lianjiang River (OLR; S5), Qiufeng River (QFR; S6), Xiashan River (XSR; S8), Zhonggang River (ZGR; S12, S13), Guitouhai River (GTR; S14), and Hucheng River (HCR; S15), as well as 8 sites in the mainstream of LRB, namely S1, S2, S9, S10, S11, S16, S17, and S18.

Surface water, and surface sediment samples (0 - 10 cm) were collected in December 2020. The locations of the sampling sites were determined by using a portable GPS (Table S1). Each surface water sample was approximately 15 liters, and the surface layer of the sediment sample was about 2 kg. They were collected in polyethylene plastic bottles or bags, marked, transported to the laboratory refrigerated, and stored at 0 - 5 °C for testing. Water samples were filtered using filter (10 cm in diameter, 0.45 μm in pore diameter, Shanghai Xin Ya Purification Equipment CO., LTD, China) as promptly as possible, and filtered water (FW), and suspended solids (SSs) retained on the filter were detected, such as total phosphorus (P), and metals. Sediment samples were dried at room temperature, homogenized, filtered through a 100-mesh sieve to obtain a powdered precipitate for each sample. The grinder, and pellet maker machine was cleaned appropriately before the next sample was prepared. P in SS, and FW samples were detected using inductively coupled plasma optical emission spectrometry (ICP-OES, Optima 8000, PerkinElmer Inc., USA), and ammonium molybdate spectrophotometry in accordance with ISO 14869-1-2001 [31], and GB/T 11893-1989 [30], respectively. Mercury (Hg), arsenic (As), and selenium (Se) in SS, and FW samples were monitored by Atomic Fluorescence Spectrometry (AFS, BAF-200C, Baode Instruments Ltd., China) in accordance with HJ 680-2013 [32], and HJ 694-2014 [33].

The pH value in the sediment was determined at a sediment: water ratio of 1:3 (w/w). The content of organic matter (OM) (g/kg) in the sediment was quantified using the chromic acid oxidation method followed by titration with ammonium ferrous sulfate in accordance with NY/T 1121.6-2006 [34]. Total Hg in the sediment was measured with a direct mercury analyzer (DMA, BDHg-60, Baode Instruments Ltd., China) in accordance with HJ 923-2017 [35]. HJ 680-2013 [32] was utilized to monitor the total As, and Se in the sediment by atomic fluorescence spectrometry (AFS, BAF-200C, Baode Instruments Ltd., China). Cd in SS, and sediment samples was analyzed with graphite furnace atomic absorption spectrophotometry (GFAS, PinAAcle 900T, PerkinElmer Inc., USA) in accordance with GB/T 17141-1997 [36]. Other total metals in FW, SS (sediment) samples were prepared in accordance with HJ 776-2015 [38], HJ 700-2014 [37], and ISO 14869-1-2001 [31] using an automatic graphite digestion (Deena2 or Deena3, Thomas Cain Inc., USA). The concentration of metals in the digests was examined by ICP-OES, and inductively coupled plasma mass spectrometer (ICP-MS, XSERIES II, Thermo Fisher Scientific Inc., USA) in accordance with EPA Method 6010D [39], and EPA Method 6020B [40]. All the reported OM, and metal concentrations in the sediments were normalized on a dry weight basis except for pH.

Quality assurance, and quality control (QA/QC), including method blanks, three-parallel samples, and standard reference materials, were carried out along with sample analyses to guarantee the analytical quality. A series of water, and soil standards, such as GSB-series environmental reference materials (Institute for Environmental Reference Materials, Ministry of Environmental Protection of China), and the ASA-series available, and the GSS-series geochemical reference materials (Institute of Geophysical, and Geochemical Prospecting, Langfang, China), were used. To ensure the accuracy of the analysis results, an average of every 10 samples was randomly selected as a parallel sample during the analysis process.

The accuracy of SSD analysis was assessed by using recoveries of standard samples with routine ranges from 75.0%-84.1% for Fe, and Al, 87.6%-107.4% for P, Ti, Mn, Mo, Be, Co, Sn, Pb, Cu, Zn, Ni, and Cr, 113.7%-115.8% for As, and 128.0%-127.5% for Sb in sediment; and in SS were 83.7%-114.9% for Al, Ti, P, Cu, Cr, and Be, 92.4%-104.4% for Mn, Pb, Ni, Sn, Co, and As, 106.0%-110.1% for Fe, Zn, and Mo, and 127.3%-129.1% for Sb; and in FW were 81.8%-120% for P, and metals. The relative deviations among the results of parallel samples were less than 20%.

Table S1 *Physical characterization of surface water samples*

Sites	Longitude	Latitude	Collection Date	Collection Time	Tem. (ºC)	pH	DO (mg/L O_2)	Con. (μs/cm)	Sality (‰)
T1	116.3493310	23.3470560	2020-12-02	13:05	22.7	8.23	8.21	653	
T2	116.3634270	23.3327760	2020-12-02	14:05	22.4	7.80	7.70	672	
T3	116.3823730	23.3221230	2020-12-02	15:05	23.1	8.31	8.50	708	
T4	116.3926770	23.3511340	2020-12-02	10:50	21.5	7.54	4.25	373	
T5	116.4077500	23.3202360	2020-12-02	11:24	23.8	6.93	7.30	708	
T6	116.4180230	23.3005030	2020-12-02	16:00	22.9	8.44	9.40	880	
T7	116.4389330	23.2897010	2020-12-02	16:30	22.5	8.70	8.80	1496	
S1	116.3147200	23.3049690	2020-12-28	10:50	21.6	7.47	4.70	667	
S2	116.3152150	23.3040030	2020-12-28	11:00	21.8	7.48	4.80	668	
S3	116.3414694	23.2959444	2020-12-28	11:55	21.7	8.20	8.44	625	
S4	116.3417690	23.2888610	2020-12-28	11:35	21.0	8.20	6.73	547	
S5	116.3519750	23.2955220	2020-12-28	12:18	20.5	7.54	5.02	676	
S6	116.3756170	23.2773580	2020-12-28	9:45	20.8	9.44	11.30	618	
S8	116.4536110	23.2677780	2020-12-28	15: 22	21.7	7.22	8.71	685	
S9	116.4832530	23.2626754	2020-12-28	10: 55	20.0	8.00	7.83	1182	
S10	116.4827747	23.2620496	2020-12-28	10: 18	20.1	7.99	8.49	1162	
S11	116.4822820	23.2613780	2020-12-28	9: 30	20.0	7.63	9.17	1391	
S12	116.4586000	23.2219000	2020-12-28	14: 45	21.5	7.83	8.14	477	
S13	116.5170330	23.2208760	2020-12-28	14: 05	21.4	8.96	9.77	3771	1.99
S14	116.5762140	23.2247080	2020-12-28	11: 42	19.4	8.04	8.24	5080	2.74
S15	116.5863700	23.2443700	2020-12-28	12: 02	21.2	7.16	7.07	2581	1.40
S16	116.6193040	23.2138380	2020-12-28	10: 52	18.9	8.13	7.82	22348	13.50
S17	116.6173680	23.2136120	2020-12-28	10: 45	18.9	8.13	8.57	20304	12.17
S18	116.6155230	23.2132650	2020-12-28	10: 01	18.7	8.46	7.22	23260	14.10
Class I for drinking and National Nature Reserve [51]						6-9	≥7.5		
Class II for drinking and National Nature Reserve [51]						6-9	≥6		
Class III [51]						6-9	≥5		
Class IV [51]						6-9	≥3		
Class V [51]						6-9	≥2		

Notes: rising tide, 05:03-13:36; falling tide, 13:36-17:02.

Table S2 *Enrichment levels by Sutherland[72] and Loska et al. [73]*

Enrichment factor	Levels
$EF < 2$	minimal
$2 \leq EF < 5$	moderate
$5 \leq EF < 20$	significant
$20 \leq EF \leq 40$	very high
$EF > 40$	extremely high

Table S3 *Risk levels by Håkanson[45]*

Single risk	Potential ecological risk index	Levels
$E_i < 40$	RI < 150	low
$40 \leq E_i < 80$	$150 \leq$ RI < 300	moderate
$80 \leq E_i < 160$	$300 \leq$ RI < 600	considerable
$160 \leq E_i < 320$	--	high
$E_i \geq 320$	RI ≥ 600	very high

Table S4 *Results of dissolved element contents in surface water*

Sites	As	Cu	Pb	Ni	Sb	Co	Cr	Zn	Mo	Sn	Ti	Mn	Fe	Al	Cd	P	Se	Hg	Be
T1	0.67	5.65	4.46	3.88	22.0	0.32	1.05	5.35	3.94	4.37	ND	0.025	ND	ND	ND	0.052	ND	ND	ND
T2	0.51	8.65	2.02	9.03	9.13	0.30	2.30	5.76	2.38	0.66	0.001	0.049	0.023	ND	0.07	0.030	ND	ND	ND
T3	0.50	6.28	2.79	9.44	0.77	0.33	1.79	4.82	2.17	0.20	0.001	0.063	ND	ND	ND	0.041	ND	ND	ND
T4	0.36	2.28	4.35	1.27	ND	0.22	3.84	7.22	0.83	0.08	0.002	0.151	0.022	ND	ND	0.371	ND	ND	ND
T5	ND	6.19	3.40	9.30	ND	0.93	2.13	16.2	1.90	0.18	0.002	0.189	ND	ND	ND	0.286	ND	ND	ND
T6	ND	4.21	1.35	8.03	ND	0.65	3.30	5.57	2.18	0.10	0.001	0.131	ND	ND	ND	0.093	ND	ND	ND
T7	0.40	4.97	1.33	6.38	11.6	0.35	5.14	4.24	2.84	ND	ND	0.099	ND	ND	ND	0.020	ND	ND	ND
S1	1.28	1.62	2.57	3.63	3.24	0.58	1.89	9.30	2.00	0.14	0.002	0.142	0.009	ND	ND	0.132	ND	ND	ND
S2	1.25	2.48	2.36	2.61	3.84	0.58	1.80	2.15	2.30	0.24	0.002	0.138	0.011	ND	ND	0.117	ND	ND	ND
S3	0.88	3.13	1.69	3.07	ND	0.29	1.68	1.69	1.91	0.31	0.002	0.007	ND	ND	ND	0.055	ND	ND	ND
S4	ND	4.07	1.66	2.60	3.36	0.16	1.82	ND	2.28	0.15	0.002	ND	ND	ND	ND	0.019	ND	ND	ND
S5	0.78	3.00	0.86	6.94	13.7	0.19	2.29	2.34	2.40	0.21	0.002	ND	ND	0.032	ND	0.034	ND	ND	ND
S6	ND	2.46	1.79	3.67	4.22	0.28	2.95	8.23	2.31	0.48	0.002	ND	0.020	0.036	ND	0.018	ND	ND	ND
S8	ND	6.42	1.96	6.38	ND	0.24	2.10	41.3	1.64	0.17	0.002	0.089	ND	ND	0.11	0.093	0.52	ND	ND
S9	0.47	1.47	1.64	5.38	6.13	0.18	1.74	3.12	2.57	0.27	0.002	0.007	ND	ND	ND	0.048	ND	ND	ND
S10	0.52	0.90	1.03	5.29	6.75	0.17	1.94	4.71	2.53	0.22	0.002	0.005	ND	ND	ND	0.027	ND	ND	ND
S11	0.52	1.41	1.91	5.05	6.46	0.18	1.73	3.94	2.48	0.37	0.002	0.017	ND	ND	ND	0.026	ND	ND	ND
S12	ND	0.20	1.80	1.28	ND	0.29	1.24	4.96	1.50	0.44	0.002	0.136	ND	ND	ND	ND	ND	ND	ND
S13	1.58	3.07	0.71	3.58	2.85	0.75	2.37	2.93	5.21	0.09	0.002	ND	ND	ND	ND	0.246	ND	ND	ND
S14	0.58	1.44	1.02	4.60	15.9	0.37	2.51	3.32	4.13	0.36	0.002	0.057	ND	ND	ND	0.054	ND	ND	ND
S15	1.41	0.68	0.87	3.10	ND	0.35	2.10	8.63	3.18	0.15	0.003	0.013	ND	ND	ND	0.297	ND	ND	ND
S16	0.89	2.40	1.14	6.99	4.05	0.73	1.99	5.21	7.89	0.34	0.005	0.068	0.016	ND	ND	0.082	ND	ND	ND
S17	0.81	2.93	0.50	6.71	3.97	0.65	1.94	2.98	7.73	0.27	0.005	0.070	ND	ND	ND	0.082	ND	ND	ND
S18	0.90	3.50	0.90	7.00	3.73	0.68	2.17	2.58	8.07	0.13	0.005	0.087	ND	ND	ND	0.060	ND	ND	ND

Notes: "ND" indicates that the concentration of pollutants are less than the corresponding detect limitations; The concerntion of Ti, Mn, Fe, Al, and P are in mg/L, for others in μg/L.

Table S5 *Statistical results of dissolved elemental contents in surface water*

Target	LLD	Unit	Mean	Minimum	Maximum	S.D.	C.V.	Limits of Class I, or central drinking-water source area [51]
As	0.30	μg L^{-1}	0.600	ND	1.580	0.473	78.83	50
Be	0.04	μg L^{-1}	ND	ND	ND	n.a.	n.a.	2
Cd	0.05	μg L^{-1}	ND	ND	0.110	0.026	260.00	1
Co	0.03	μg L^{-1}	0.410	0.160	0.930	0.224	54.63	1000
Cr	0.11	μg L^{-1}	2.240	1.050	5.140	0.858	38.30	10
Cu	0.08	μg L^{-1}	3.310	0.200	8.650	2.130	64.35	10
Hg	0.015	μg L^{-1}	ND	ND	ND	n.a.	n.a.	0.05
Mo	0.06	μg L^{-1}	3.180	0.830	8.070	2.030	63.84	70
Ni	0.06	μg L^{-1}	5.220	1.270	9.440	2.421	46.38	20
Pb	0.09	μg L^{-1}	1.840	0.500	4.460	1.056	57.39	10
Sb	0.30	μg L^{-1}	5.070	ND	22.000	5.746	113.33	5
Se	0.30	μg L^{-1}	0.020	ND	0.520	0.106	530.00	10
Sn	0.08	μg L^{-1}	0.410	ND	4.370	0.855	208.54	n.a.
Zn	0.67	μg L^{-1}	6.520	ND	41.300	8.100	124.23	50
P	0.015	mg L^{-1}	0.095	ND	0.371	0.101	106.32	0.02
Ti	0.001	mg L^{-1}	0.002	ND	0.005	0.001	50.00	0.1
Mn	0.001	mg L^{-1}	0.064	ND	0.189	0.059	92.19	0.1
Fe	0.009	μg L^{-1}	0.004	ND	0.023	0.008	200.00	0.3
Al	0.009	mg L^{-1}	0.003	ND	0.036	0.010	333.33	n.a.

Notes: "ND" indicates that the concentration of pollutants are less than the corresponding detect limitations, and zero were used for statistics; "S.D." standard deviation; "C.V." coefficient of variation (%); "n.a." presented that the values are not available.

Table S6 *Results of particulate-phase elemental contents in surface water*

Sites	SS	Al	Ti	Co	Fe	Mn	Hg	Zn	Cu	Pb	As	Be	Cr	Ni	Se	Mo	Cd	P	Sn	Sb
T1	68.7	94.070	4.643	15.90	38.728	1.514	0.346	198.84	114.70	90.76	9.34	5.85	57.21	38.28	0.96	2.22	0.385	1.107	46.01	8.68
T2	23.9	148.203	5.460	57.02	47.477	5.235	0.984	533.05	663.60	231.00	13.84	5.87	87.45	166.53	1.53	3.74	3.630	2.657	272.25	48.39
T3	20.6	93.075	4.319	42.18	38.285	6.034	0.578	348.54	321.36	117.60	6.82	4.41	71.84	141.26	1.03	2.67	1.757	2.936	89.43	15.46
T4	23.8	44.949	2.445	25.36	38.253	11.132	0.189	294.12	55.88	46.95	3.37	1.71	36.55	27.31	1.18	1.91	0.845	10.044	15.05	1.59
T5	5.5	82.391	3.575	70.03	38.644	0.944	0.878	405.45	83.64	42.96	4.72	2.28	110.91	58.18	0.50	1.63	0.515	3.111	11.47	2.58
T6	7.7	63.348	2.495	134.78	21.970	14.757	0.158	511.69	98.70	53.07	2.64	1.96	85.71	116.88	0.85	2.56	1.810	3.721	22.01	6.55
T7	26.1	98.200	5.357	19.34	52.323	3.420	0.480	396.93	214.18	105.28	7.92	4.60	73.18	74.33	1.70	1.98	1.157	4.559	64.18	10.80
S1	26.2	69.489	3.840	31.07	24.914	11.234	0.203	309.30	60.93	93.64	13.19	3.05	64.65	34.42	1.12	2.39	0.967	4.182	25.38	6.29
S2	21.5	55.232	3.031	27.54	23.494	9.086	0.131	264.12	63.36	79.32	11.52	3.03	58.02	29.01	1.02	2.03	0.718	3.877	22.79	5.71
S3	13.7	43.186	2.114	20.05	21.999	10.523	0.170	351.09	70.80	98.83	12.42	2.56	40.88	56.93	2.27	3.59	0.982	5.773	75.97	7.04
S4	22.9	100.175	4.262	12.83	34.732	2.028	0.292	319.74	135.53	167.72	7.27	3.58	74.12	44.30	1.25	2.01	1.034	2.202	110.45	7.22
S5	20.8	76.196	3.158	17.02	26.672	4.640	0.544	453.85	381.73	201.54	14.08	2.92	69.23	109.13	1.42	2.19	2.630	2.611	156.40	33.76
S6	27.3	86.172	3.382	12.40	29.656	1.154	0.544	260.07	76.19	59.44	4.79	2.88	50.55	42.49	0.92	1.20	0.399	2.668	36.22	6.07
S8	12.3	70.262	2.878	29.74	31.598	11.111	0.436	1662.60	278.05	56.37	6.78	2.40	132.52	79.67	4.70	4.27	5.089	4.864	61.27	5.69
S9	17.3	27.836	1.400	10.22	14.460	6.397	0.138	249.71	55.49	56.14	5.56	1.68	20.81	61.27	1.75	1.91	0.468	3.336	25.54	8.32
S10	13.7	29.788	1.553	10.52	16.204	7.983	0.150	262.04	62.77	60.24	6.29	1.83	20.44	72.26	2.21	2.27	0.701	3.574	48.14	10.25
S11	18.3	60.995	2.476	13.41	23.743	7.597	0.294	356.82	142.05	76.74	8.93	2.86	48.86	78.41	1.98	2.61	1.220	3.428	51.51	15.79
S12	67.8	40.986	2.098	6.89	21.983	1.923	0.934	134.07	17.40	28.21	3.18	1.56	29.65	13.86	0.43	0.74	0.207	1.095	28.90	3.52
S13	74.4	73.795	3.098	12.20	32.112	2.576	0.477	161.02	27.69	47.88	6.82	2.93	47.58	32.12	0.67	1.02	0.224	3.787	15.28	2.17
S14	27.1	57.294	2.801	12.55	30.413	3.396	0.256	170.85	46.86	44.13	9.21	2.43	50.55	37.27	1.18	1.41	0.481	1.682	26.27	8.40
S15	9.5	44.346	2.316	10.22	20.846	0.545	0.651	272.63	38.95	57.78	6.80	1.73	44.21	48.42	0.84	1.41	0.596	3.951	68.51	1.07
S16	35.0	56.477	1.974	7.25	19.488	0.788	0.094	98.57	16.29	38.51	4.03	1.95	26.00	15.71	0.37	0.92	0.100	0.499	33.46	1.15
S17	35.2	69.097	2.385	8.42	23.363	0.888	0.110	114.77	19.03	44.33	4.67	2.35	26.99	19.60	0.42	1.10	0.106	0.698	9.55	1.11
S18	37.3	62.806	2.923	12.27	25.518	1.035	0.113	124.66	21.98	66.10	5.65	2.40	37.80	20.64	0.49	1.18	0.139	0.692	12.78	1.38

Notes: "SS" refers to the concentration of suspended solids in surface water; the concerntion of SS, Ti, Mn, Fe, Al, and P are in g/kg, for others in μg/kg.

Table S7 *Statistical results of particulate-phase elemental content in surface water*

Target	MDL	Unit	Mean	Minimum	Maximum	S.D.	C.V. (%)
As	0.05	mg/kg	7.420	2.640	14.330	3.230	43.53
Be	0.01	mg/kg	2.870	1.560	5.870	1.210	42.16
Cd	0.010	mg/kg	0.880	0.000	4.800	1.170	132.95
Co	0.04	mg/kg	25.800	6.890	134.780	28.080	108.84
Cr	0.90	mg/kg	56.900	20.400	132.300	27.990	49.19
Cu	0.50	mg/kg	127.700	16.300	663.400	150.880	118.15
Hg	0.002	mg/kg	0.311	0.062	0.833	0.236	75.88
Mo	0.05	mg/kg	2.040	0.740	4.270	0.900	44.12
Ni	0.50	mg/kg	59.100	13.800	166.500	40.100	67.85
Pb	0.50	mg/kg	81.900	28.200	231.000	51.800	63.25
Sb	0.08	mg/kg	9.120	1.070	48.390	10.890	119.41
Se	0.010	mg/kg	1.340	0.440	4.420	0.840	62.69
Sn	0.10	mg/kg	55.370	9.550	272.250	58.140	105.00
Zn	0.90	mg/kg	343.800	98.500	1662.700	305.710	88.92
P	0.005	g/kg	3.211	0.499	10.044	2020	62.91
Mn	0.005	g/kg	5.247	0.545	14.756	4255	81.09
Ti	0.005	g/kg	3.083	1.400	5.460	1098	35.61
Fe	0.010	g/kg	29.037	14.460	52.323	9556	32.91
Al	0.010	g/kg	68.682	27.836	148.203	26655	38.81

Table S8 *Results of pH, organic matter (OM), phosphorus (P), and metal contents in surficial channel sediments*

Sites	Al	Fe	Ti	P	Mn	As	Be	Cd	Co	Cr	Cu	Hg	Mo	Ni	Pb	Sb	Se	Sn	Zn	OM	pH
T1	93.764	35.445	3.9	0.416	0.567	8.47	4.61	0.18	11.68	59.76	85.51	0.403	3.01	24.89	60.82	6.94	0.529	30.68	105.35	27.6	7.23
T2	105.02	28.424	3.784	1.317	0.557	10.93	4.81	1.387	12.63	58.14	246.93	0.628	2.01	40.17	135.97	30.58	0.747	103.44	238.57	43.5	7.86
T3	102.05	34.506	3.585	1.604	0.605	11.72	5.72	2.514	16.98	64.77	1097.84	1.528	2.93	112.28	223.26	75.87	0.503	227.21	355.34	57.9	7.53
T4	89.071	28.705	3.546	0.801	0.508	4.58	3.16	0.718	9.55	121.15	58.19	0.202	1.66	93.61	42.69	2.23	0.435	28.61	170.33	33.0	8.10
T5	89.381	37.155	4.097	1.702	0.715	5.17	3.32	0.431	15.48	184.96	116.14	0.244	2.78	37.17	47.24	14.37	0.601	81.69	297.93	70.5	7.56
T6	93.116	33.043	4.357	1.00	0.757	8.28	4.00	0.571	15.71	76.35	210.13	0.298	1.66	45.07	73.52	13.1	0.503	56.53	196.01	30.6	7.42
T7	110.416	44.726	4.803	0.747	0.626	4.91	6.46	0.511	15.12	56.06	101.86	0.137	1.58	29.28	49.06	4.69	0.502	26.14	177.82	25.5	7.98
S1	96.029	26.720	3.456	0.883	0.566	19.56	4.89	1.39	9.22	68.53	477.52	0.114	2.22	29.63	89.58	10.61	0.842	99.88	287.58	40.6	7.53
S2	123.587	17.381	3.688	0.214	0.203	6.01	2.73	0.386	6.01	29.28	25.29	0.225	1.03	12.13	97.84	0.78	1.008	26.72	74.76	16.3	8.05
S3	98.931	33.135	3.772	0.713	0.675	10.41	5.27	0.496	13.65	55.41	50.52	0.178	1.74	21.40	61.63	2.25	0.56	25.38	161.26	27.0	7.97
S4	81.751	31.156	3.07	0.882	0.607	6.94	3.48	0.682	9.31	51.28	184.56	0.709	1.63	28.82	96.92	4.55	0.562	72.55	206.7	30.2	7.99
S5	92.951	35.111	4.125	0.258	0.439	10.07	5.09	0.417	17.7	68.29	18.76	0.068	1.79	33.30	49.34	1.03	0.409	10.89	128.46	26.8	7.90
S6	90.214	30.032	3.585	0.54	0.404	6.83	3.25	0.452	9.47	67.17	157.23	0.310	1.7	48.66	57.50	4.69	0.503	45.63	178.91	25.9	8.25
S8	88.966	33.471	3.207	1.394	0.567	7.38	3.99	10.888	12.26	95.53	194.44	0.95	3.32	62.58	84.01	24.07	0.848	179.91	419.93	93.7	7.75
S9	92.678	31.925	3.329	1.86	0.525	17.7	4.75	1.709	12.65	87.23	237.62	0.636	3.18	55.83	112.51	25.56	1.209	163.15	340.44	84.2	7.94
S11	113.295	35.558	4.348	1.037	0.427	20.62	5.32	1.486	15.24	87.42	286.24	0.424	3.73	66.10	85.99	30.08	1.019	129.64	351.27	89.3	7.94
S12	84.851	36.844	3.211	1.949	0.703	5.11	3.52	1.042	12.92	60.16	52.12	0.641	3.54	23.62	61.30	5.39	0.78	159.34	287.74	69.2	8.89
S13	58.11	22.77	2.617	0.525	0.364	7.39	5.77	0.165	8.91	36.24	19.76	0.184	1.09	15.21	36.36	1.57	0.299	17.81	90.81	34.5	8.34
S14	39.406	15.999	1.639	0.538	0.353	5.69	1.84	0.348	6.97	25.54	13.41	0.076	0.56	12.15	26.06	1.52	0.214	6.99	67.51	38.5	7.81
S15	88.475	32.274	3.67	2.867	0.448	10.06	3.46	1.352	10.81	106.15	126.83	2.063	3.29	31.97	115.03	2.27	1.131	405.28	669.52	112	7.33
S16	63.267	23.663	2.264	0.263	0.425	8.17	2.64	0.626	8.11	29.97	17.29	0.072	0.82	13.64	40.53	0.54	0.293	16.01	70.56	13.5	8.63
S17	64.845	26.25	2.671	0.34	0.444	6.23	2.9	0.355	8.41	34.54	23.54	0.084	1.18	14.83	41.30	0.66	0.274	23.44	88.87	19.9	8.74
S18	84.869	30.088	3.068	0.775	0.314	12.59	3.79	1.541	11.75	68.3	191.16	0.337	4.09	47.12	74.24	12.62	0.816	69.92	306.96	78.3	8.50

Notes: OM, Al, Fe, Ti, Mn, and P in g kg^{-1}, others in mg kg^{-1} (except for pH value).

Table S9 *Statistical results of pH, organic matter (OM), phosphorus (P), and metal contents in surficial channel sediments*

Targets	LLD	Unit	Mean	Min.	Max.	S.D.	C.V.	Background value [44]	Standard Grade I [52]
pH	n.a.	n.a.	7.970	7.230	8.890	0.440	5.520	n.a.	n.a.
OM	0.13	g/kg	47.330	13.500	112.000	28.320	59.840	n.a.	n.a.
As	0.01	mg/kg	9.500	3.810	19.600	4.800	50.530	12.5	20.0
Be	0.05	mg/kg	4.120	1.840	6.460	1.180	28.640	2.2	n.a.
Cd	0.01	mg/kg	0.560	0.030	4.300	0.890	158.930	0.219	0.50
Co	0.04	mg/kg	11.800	6.010	17.700	3.300	27.970	13.5	n.a.
Cr	2.0	mg/kg	69.200	25.500	185.000	35.200	50.870	70.0	80.0
Cu	0.5	mg/kg	173.600	13.400	1097.800	231.100	133.120	25.0	35.0
Hg	0.002	mg/kg	0.457	0.068	2.063	0.491	107.440	0.072	0.20
Mo	0.05	mg/kg	2.200	0.560	4.090	1.000	45.450	1.02	n.a.
Ni	0.5	mg/kg	39.100	12.100	112.300	25.700	65.730	29.0	n.a.
Pb	0.5	mg/kg	76.600	26.100	223.300	42.800	55.870	31.0	60.0
Sb	0.08	mg/kg	14.700	0.790	88.320	20.200	137.410	1.05	n.a.
Se	0.01	mg/kg	0.520	0.200	1.070	0.250	48.080	0.31	n.a.
Sn	0.10	mg/kg	87.300	6.990	405.280	92.900	106.410	3.50	n.a.
Zn	1.0	mg/kg	229.200	67.500	669.500	142.300	62.090	81.0	150.0
P	0.005	g/kg	0.984	0.214	2.867	0.662	67.280	555	n.a.
Mn	0.005	g/kg	0.513	0.203	0.757	0.140	27.290	0.739	n.a.
Ti	0.005	g/kg	3.469	1.639	4.803	0.717	20.670	4.646	n.a.
Fe	0.010	g/kg	30.625	15.999	44.726	6.505	21.240	34.160	n.a.
Al	0.010	g/kg	88.915	39.406	123.587	18.631	20.950	72.529	n.a.

Notes: "n.a." presented that the value is not available; C.V. was calculated by the concentrations of pollutants.

Table S10 *Standards for marine sediment quality [52]*

Items	Grade I	Grade II	Grade III
Hg	0.20	0.50	1.00
Cd	0.50	1.50	5.00
Pb	60.0	130.0	250.0
Zn	150.0	350.0	600.0
Cu	35.0	100.0	200.0
Cr	80.0	150.0	270.0
As	20.0	65.0	93.0

Note: Values are in mg/kg dry wt.

Table S11 *Enrichment factors of elements detected in suspended solids (SSs) and surficial channel sediments (SCSs)*

Sites	Sn		Sb		Mn		P		Hg		Cd		Cu		Pb		Mo		Ni		Zn		Se		Be		Co		Cr		As		Al		Ti	
	SSs	SCSs	SSs	SCSs	SSs	SCSs	SSs	SCSs	SSs	SCSs	SSs	SCSs	SSs	SCSs	SSs	SCSs	SSs	SCSs	SSs	SCSs	SSs	SCSs	SSs	SCSs	SSs	SCSs	SSs	SCSs	SSs	SCSs	SSs	SCSs	SSs	SCSs	SSs	SCSs
T1	11.6	8.4	7.3	6.4	1.8	0.7	1.8	0.7	4.2	5.4	1.6	0.8	4.0	3.3	2.6	1.9	1.9	2.8	1.2	0.8	2.2	1.3	2.7	1.6	2.3	2.0	1.0	0.8	0.7	0.8	0.7	0.7	1.1	1.2	0.9	0.8
T2	56.0	35.5	33.2	35.0	5.1	0.9	3.4	2.9	9.8	10.5	11.9	7.6	19.1	11.9	5.4	5.3	2.6	2.4	4.1	1.7	4.7	3.5	3.6	2.9	1.9	2.6	3.0	1.1	0.9	1.0	0.8	1.1	1.5	1.7	0.8	1.0
T3	22.8	64.3	13.1	71.5	7.3	0.8	4.7	2.9	7.2	21.0	7.2	11.4	11.5	43.5	3.4	7.1	2.3	2.8	4.3	3.8	3.8	4.3	3.0	1.6	1.8	2.6	2.8	1.2	0.9	0.9	0.5	0.9	1.1	1.4	0.8	0.8
T4	3.8	9.7	1.4	2.5	13.5	0.8	16.2	1.7	2.3	3.3	3.4	3.9	2.0	2.8	1.4	1.6	1.7	1.9	0.8	3.8	3.2	2.5	3.4	1.7	0.7	1.7	1.7	0.8	0.5	2.1	0.2	0.4	0.6	1.5	0.5	0.9
T5	2.9	21.5	2.2	12.6	1.1	0.9	5.0	2.8	10.8	3.1	2.1	1.8	3.0	4.3	1.2	1.4	1.4	2.5	1.8	1.2	4.4	3.4	1.4	1.8	0.9	1.4	4.6	1.1	1.4	2.4	0.3	0.4	1.0	1.1	0.7	0.8
T6	9.8	16.7	9.7	12.9	31.0	1.1	10.4	1.9	3.4	4.3	12.9	2.7	6.1	8.7	2.7	2.5	3.9	1.7	6.3	1.6	9.8	2.5	4.3	1.7	1.4	1.9	15.5	1.2	1.9	1.1	0.3	0.7	1.4	1.3	0.8	1.0
T7	12.0	5.7	6.7	3.4	3.0	0.6	5.4	1.0	4.4	1.5	3.4	1.8	5.6	3.1	2.2	1.2	1.3	1.2	1.7	0.8	3.2	1.7	3.6	1.2	1.4	2.2	0.9	0.9	0.7	0.6	0.4	0.3	0.9	1.2	0.8	0.8
S1	9.9	15.0	8.2	1.5	20.8	0.5	10.3	0.8	3.9	6.1	6.1	3.5	3.3	2.0	4.1	6.2	3.2	2.0	1.6	0.8	5.2	1.8	5.0	6.4	1.9	2.4	3.2	0.9	1.3	0.8	1.4	0.9	1.3	3.3	1.1	1.6
S2	9.5	36.5	7.9	12.9	17.9	1.0	10.2	2.0	2.6	2.0	4.8	8.1	3.7	24.4	3.7	3.7	2.9	2.8	1.5	1.3	4.7	4.5	4.8	3.5	2.0	2.8	3.0	0.9	1.2	1.3	1.3	2.0	1.1	1.7	0.9	1.0
S3	33.7	7.5	10.4	2.2	22.1	0.9	16.2	1.3	3.7	2.5	7.0	2.3	4.4	2.1	5.0	2.0	5.5	1.8	3.0	0.8	6.7	2.1	11.4	1.9	1.8	2.5	2.3	1.0	0.9	0.8	1.5	0.9	0.9	1.4	0.7	0.8
S4	31.0	22.7	6.8	4.8	2.7	0.9	3.9	1.7	4.0	10.8	4.6	3.4	5.3	8.1	5.3	3.4	1.9	1.8	1.5	1.1	3.9	2.8	4.0	2.0	1.6	1.7	0.9	0.8	1.0	0.8	0.6	0.6	1.4	1.2	0.9	0.7
S5	57.2	3.0	41.2	1.0	8.0	0.6	6.0	0.5	9.7	0.9	15.4	1.9	19.6	0.7	8.3	1.5	2.7	1.7	4.8	1.1	7.2	1.5	5.9	1.3	1.7	2.3	1.6	1.3	1.3	0.9	1.4	0.8	1.3	1.2	0.9	0.9
S6	11.9	14.8	6.7	5.1	1.8	0.6	5.5	1.1	8.7	4.9	2.1	2.3	3.5	7.2	2.2	2.1	1.4	1.9	1.7	1.9	3.7	2.5	3.4	1.8	1.5	1.7	1.1	0.8	0.8	1.1	0.4	0.6	1.4	1.4	0.8	0.9
S8	18.9	52.5	5.9	23.4	16.3	0.8	9.5	2.6	6.5	13.5	25.1	50.7	12.0	7.9	2.0	2.8	4.5	3.3	3.0	2.2	22.2	5.3	16.4	2.8	1.2	1.9	2.4	0.9	2.0	1.4	0.6	0.6	1.0	1.3	0.7	0.7
S9	17.2	49.9	18.7	26.0	20.4	0.8	14.2	3.6	4.5	9.5	5.0	8.3	5.2	10.2	4.3	3.9	4.4	3.3	5.0	2.1	7.3	4.5	13.3	4.2	1.8	2.3	1.8	1.0	0.7	1.3	1.1	1.5	0.9	1.4	0.7	0.8
S10	29.0	n.a.	20.6	n.a.	22.8	n.a.	13.6	n.a.	4.4	n.a.	6.7	n.a.	5.3	n.a.	4.1	n.a.	4.7	n.a.	5.3	n.a.	6.8	n.a.	15.0	n.a.	1.8	n.a.	1.6	n.a.	0.6	n.a.	1.1	n.a.	0.9	n.a.	0.7	n.a.
S11	21.2	35.6	21.6	27.5	14.8	0.6	8.9	1.8	5.9	5.7	8.0	6.5	8.2	11.0	3.6	2.7	3.7	3.5	3.9	2.2	6.3	4.2	9.2	3.2	1.9	2.3	1.4	1.1	1.0	1.2	1.0	1.6	1.2	1.5	0.8	0.9
S12	12.8	42.2	5.2	4.8	4.0	0.9	3.1	3.3	20.2	8.3	1.5	4.4	1.1	1.9	1.4	1.8	1.1	3.2	0.7	0.8	2.6	3.3	2.2	2.3	1.1	1.5	0.8	0.9	0.7	0.8	0.4	0.4	0.9	1.1	0.7	0.6
S13	4.6	7.6	2.2	2.2	3.7	0.7	7.3	1.4	7.0	3.8	1.1	1.1	1.2	1.2	1.6	1.8	1.1	1.6	1.2	0.8	2.1	1.7	2.3	1.4	1.4	3.9	1.0	1.0	0.7	0.8	0.6	0.9	1.1	1.2	0.7	0.8
S14	8.4	4.3	9.0	3.1	5.2	1.0	3.4	2.1	4.0	2.3	2.5	3.4	2.1	1.1	1.6	1.8	1.6	1.2	1.4	0.9	2.4	1.8	4.3	1.5	1.2	1.8	1.0	1.1	0.8	0.8	0.8	1.0	0.9	1.2	0.7	0.8
S15	32.1	122.6	1.7	2.3	1.2	0.6	11.7	5.5	14.8	30.3	4.5	6.5	2.6	5.4	3.1	3.9	2.3	3.4	2.7	1.2	5.5	8.7	4.4	3.9	1.3	1.7	1.2	0.8	1.0	1.6	0.9	0.9	1.0	1.3	0.8	0.8
S16	16.8	6.6	1.9	0.7	1.9	0.8	1.6	0.7	2.3	1.4	0.8	4.1	1.1	1.0	2.2	1.9	1.6	1.2	0.9	0.7	2.1	1.3	2.1	1.4	1.6	1.7	0.9	0.9	0.7	0.6	0.6	0.9	1.4	1.3	0.7	0.7
S17	4.0	8.7	1.5	0.8	1.8	0.8	1.8	0.8	2.2	1.5	0.7	2.1	1.1	1.2	2.1	1.7	1.6	1.5	1.0	0.7	2.1	1.4	2.0	1.2	1.6	1.7	0.9	0.8	0.6	0.6	0.5	0.6	1.4	1.2	0.8	0.7
S18	4.9	22.7	1.8	13.6	1.9	0.5	1.7	1.6	2.1	5.3	0.8	8.0	1.2	8.7	2.9	2.7	1.5	4.6	1.0	1.8	2.1	4.3	2.1	3.0	1.5	2.0	1.2	1.0	0.7	1.1	0.6	1.1	1.2	1.3	0.8	0.7
Mean	18.4	26.7	10.2	12.0	9.6	0.8	7.3	1.9	6.2	6.9	5.8	6.4	5.5	7.5	3.2	2.8	2.5	2.4	2.5	1.5	5.2	3.1	5.4	2.4	1.6	2.1	2.3	1.0	1.0	1.1	0.8	0.9	1.1	1.4	0.8	0.8

Note: "n.a." presents that surficial channel sediment sampling at S10 was not carried out, and no relevant data are available.

Table S12 *Analysis of the main components of pollutants in suspended solids in surface water and surficial channel sediments*

Pollutants	PC 1	PC 2	PC 3	PC 4
Sb	.900	.010	.237	.120
Pb	.881	.081	.201	.089
Cu	.869	.053	.275	.138
As	.687	.275	-.041	-.365
Se	.066	.932	.048	.076
Zn	.063	.779	.480	.210
Mn	-.018	.651	-.274	.592
Mo	.397	.630	.448	.037
Hg	.377	-.113	.767	-.019
Cr	.014	.248	.719	.190
Sn	.574	.090	.683	-.137
Cd	.252	.394	.528	.080
Co	.014	.123	.113	.903
Ni	.573	.269	.226	.614
Eigenvalue	3.844	2.706	2.596	1.831
Variance %	27.455	46.780	65.322	78.398

Table S13 *The potential ecological risk assessment for suspended solids in surface water*

Sites		Hg	Cd	Sn	Sb	Cu	Mo	Pb	Ni	Co	Be	Mn	As	Zn	Cr	V	Ti	RI
T2		547	497	311	230	133	48	37	29	21	16	7	11	7	2	2	1	1766
S8		242	697	70	27	56	54	9	14	11	7	15	5	21	4	1	1	1146
S5		302	360	179	161	76	28	33	19	6	8	6	11	6	2	1	1	1106
T7		267	158	73	51	43	25	17	13	7	13	5	6	5	2	2	1	1062
T3		321	241	102	74	64	34	19	24	16	12	8	5	4	2	2	1	836
T5		488	71	13	12	17	21	7	10	26	6	1	4	5	3	1	1	622
S12		519	28	33	17	3	9	5	2	3	4	3	3	2	1	1	0	609
S15		362	82	78	5	8	18	9	8	4	5	1	5	3	1	1	0	553
S11		163	167	59	75	28	33	12	14	5	8	10	7	4	1	1	1	525
S4		162	142	126	34	27	26	27	8	5	10	3	6	4	2	1	1	517
S6		302	55	41	29	15	15	10	7	5	8	2	4	3	1	1	1	457
T6		88	248	25	31	20	33	9	20	50	5	20	2	6	2	1	1	445
S3		94	135	87	34	14	46	16	10	7	7	14	10	4	1	1	0	410
T1		192	53	53	41	23	28	15	7	6	16	2	7	2	2	2	1	390
S1		113	132	29	30	12	30	15	6	12	8	15	11	4	2	1	1	346
S13		265	31	17	10	6	13	8	6	5	8	3	5	2	1	1	1	342
S10		83	96	55	49	13	29	10	12	4	5	11	5	3	1	1	0	325
S14		142	66	30	40	9	18	7	6	5	7	5	7	2	1	1	1	305
T4		105	116	17	8	11	24	8	5	9	5	15	3	4	1	1	1	281
S2		73	98	26	27	13	26	13	5	10	8	12	9	3	2	1	1	263
S9		77	64	29	40	11	24	9	11	4	5	9	4	3	1	1	0	245
S16		52	14	38	5	3	12	6	3	3	5	1	3	1	1	1	0	124
S18		63	19	15	7	4	15	11	4	5	7	1	5	2	1	1	1	123
S17		61	15	11	5	4	14	7	3	3	6	1	4	1	1	1	1	110
	Toxic response factor	40	30	4	5	5	13	5	5	5	6	1	10	1	2	2	1	--
E_r^i	Min	52	14	11	5	3	9	5	2	3	4	1	2	1	1	1	0	110
	Max	547	697	311	230	133	54	37	29	50	16	20	11	21	4	2	1	1766
	Median	163	97	40	31	14	26	10	8	6	7	6	5	4	1	1	1	428
	Mean	212	149	63	43	26	26	13	10	10	8	7	6	4	2	1	1	538

Notes: The toxic-response factor of As, Cd, Cr, Cu, Hg, Pb, and Zn were calculated by Håkanson [45], Co, Mn, Ni, and Ti by Xu *et al.* [46], Be and Sb by Wang *et al.* [48], Sn and Mo by Aksu *et al.*[47].

Table S14 *The potential ecological risk assessment for surficial channel sediments in the Lianjiang River Basin*

Sites	Hg	Cd	Sn	Sb	Cu	Mo	Pb	Be	Ni	As	Co	Zn	Cr	Mn	V	Ti	RI
S8	528	1492	206	115	39	42	14	11	11	6	5	5	3	1	1	1	2494
T3	849	344	260	361	220	37	36	16	19	9	6	4	2	1	1	1	2189
S15	1146	185	463	11	25	42	19	9	6	8	4	8	3	1	1	1	1948
S9	353	234	186	122	48	41	18	13	10	14	5	4	2	1	1	1	1071
T2	349	190	118	146	49	26	22	13	7	9	5	3	2	1	1	1	962
S11	236	204	148	143	57	48	14	15	11	16	6	4	2	1	2	1	929
S12	356	143	182	26	10	45	10	10	4	4	5	4	2	1	1	1	819
S4	394	93	83	22	37	21	16	9	5	6	3	3	1	1	1	1	710
S18	187	211	80	60	38	52	12	10	8	10	4	4	2	0	1	1	695
S1	63	190	114	51	96	28	14	13	5	16	3	4	2	1	1	1	625
T6	166	78	65	62	42	21	12	11	8	7	6	2	2	1	2	1	500
T5	136	59	93	68	23	35	8	9	6	4	6	4	5	1	1	1	474
T1	224	25	35	33	17	38	10	13	4	7	4	1	2	1	2	1	435
S6	172	62	52	22	31	22	9	9	8	5	4	2	2	1	1	1	416
T4	112	98	33	11	12	21	7	9	16	4	4	2	3	1	1	1	348
S3	99	68	29	11	10	22	10	14	4	8	5	2	2	1	1	1	307
T7	76	70	30	22	20	20	8	18	5	4	6	2	2	1	2	1	303
S2	125	53	31	4	5	13	16	7	2	5	2	1	1	0	1	1	285
S13	102	23	20	7	4	14	6	16	3	6	3	1	1	0	1	1	218
S5	38	57	12	5	4	23	8	14	6	8	7	2	2	1	2	1	205
S16	40	86	18	3	3	10	7	7	2	7	3	1	1	1	1	0	202
S17	47	49	27	3	5	15	7	8	3	5	3	1	1	1	1	1	187
S14	42	48	8	7	3	7	4	5	2	5	3	1	1	0	1	0	147
Toxic response factor*	40	30	4	5	5	13	5	6	5	10	5	1	2	1	2	1	--
E_r^i Min	38	23	8	3	3	7	4	5	2	4	2	1	1	0	1	0	147
E_r^i Max	1146	1492	463	361	220	52	36	18	19	16	7	8	5	1	2	1	2494
E_r^i Median	169	86	65	22	23	23	10	11	6	7	4	2	2	1	1	1	474
E_r^i Mean	261	177	100	57	35	28	12	11	7	8	4	3	2	1	1	1	716

Notes: The toxic-response factor of As, Cd, Cr, Cu, Hg, Pb, and Zn were calculated by Håkanson [45], Co, Mn, Ni, and Ti by Xu *et al.* [46], Be and Sb by Wang *et al.* [48], Sn and Mo by Aksu *et al.*[47]

Table S15 *Comparison of pollutant concentrations in surficial channel sediments in rivers in Guiyu Town, China*

Rivers	Sampling date	Sb	Sn	Cu	Hg	Cd	Zn	Pb	Mo	Be	Ni	Cr	Co	As	Mn	References
Bergang River in Guiyu Town in China	2020.12	22.82±29.49	70.65±70.53	241.9±354.0	0.439±0.473	0.42±0.38	208.7±84.5	85.2±63.5	2.18±0.62	4.65±1.14	52.0±32.5	86.2±45.1	14.36±2.80	8.919±4.281	0.597±0.104	In this study
		1.46-88.32	10.89-227.21	18.8-1097.8	0.068-1.528	0.06-1.24	105.4-355.3	42.7-223.3	1.58-3.01	3.16-6.46	24.9-112.3	56.1-185	9.55-17.7	4.86-17.70	0.439-0.757	
Rivers in Guiyu Town in China	2021.4	n.a.	n.a.	733±926	n.a.	1.40±0.95	325±230	137.0±59.1	n.a.	n.a.	62.9±31.4	62.6±18.2	10.60±2.28	n.a.	n.a.	[27]
		n.a.	n.a.	67.4-3077	n.a.	0.51-3.33	108-758	80.6-268.0	n.a.	n.a.	20.0-114.0	38.5-94.4	6.00-14.60	n.a.	n.a.	
Old Lianjiang River in Guiyu Town in China	2018.01	362.6	366.2	3025.8	n.a.	8.21	1534	1343.6	n.a.	n.a.	389.2	146.4	20.36	n.a.	n.a.	[26]
An abandoned E-waste burning Site, and neighboring stream sites in Guiyu Town in China	2014.7	210.3	502.84	527.25	n.a.	1.50	n.a.	231.80	2.45	3.46	51.3	48.33	9.09	10.37	0.305	[25]
		15.5-470.0	22.50-1870	17.10-166	n.a.	0.02-3.72	n.a.	20.50-744	1.49-4.24	2.50-4.80	15.40-137.50	35-65	5.9-16.7	2.7-25.2	0.173-0.587	
Bergang River in Guivu Town in China	2013.5	3990	n.a.	3370	n.a.	5.66	1190	2170	n.a.	1.18	216	10.2	7.66	n.a.	0.100	[24]
Tributaries, and mainstream of Lianang River in Guiyu Town in China (Guiyu Town region)	2013.1	225	542	4000	1.43	17.7	1430	1140	14.5	1.13	623	114	40.2	24.5	0.452	[21]
		25.1-632	57.0-1940	93.8-18500	0.24-3.17	0.55-81.0	206-4480	87.8-3930	3.94-35.5	0.057-2.66	30.5-1900	35.4-326	8.31-114	13.2-39.2	0.200-0.692	
Bergang River in Guiyu Town in China	2006.4	n.a.	n.a.	66.7±82.3	n.a.	0.24±0.25	133.7±26.7	55±20.8	n.a.	n.a.	51.5±60.8	35.3±11.4	n.a.	n.a.	n.a.	[23]
		n.a.	n.a.	12.4-217	n.a.	ND-0.55	106-182	39.7-96.6	n.a.	n.a.	20.8-175	26.7-52.5	n.a.	n.a.	n.a.	
Bergang River in Guiyu Town in China	2005.8, 2005.9	n.a.	n.a.	65.1±101	n.a.	n.a.	107±54.9	47.3±13.8	n.a.	n.a.	25.2±10.5	n.a.	12.7±3.63	n.a.	n.a.	[22]
		n.a.	n.a.	17.0-346	n.a.	ND-0.57	51.3-249	28.6-76.9	n.a.	n.a.	12.4-39.8	n.a.	9.10-20.1	n.a.	n.a.	
Bergang River in Guiyu Town in China	2005.10	n.a.	n.a.	1839.3±2381.6	0.638±0.155	1.010±0.488	n.a.	183.7±79.80	n.a.	n.a.	446.3±775.4	n.a.	n.a.	11.5±3.21	n.a.	[19]
		n.a.	n.a.	73.0-9962	0.331-0.980	0.451-2.38	n.a.	29.0-274	n.a.	n.a.	24.0-3059	n.a.	n.a.	6.38-19.6	n.a.	
Old lianang River in Guivu Town in China	2015, 2016	n.a.	n.a.	252±628	0.631	1.24±0.62	272±169	112±70.9	n.a.	n.a.	39.1	52.7±40.1	17.8	17.5±13.3	0.437	[28]
Old lianang River in Guiyu Town in China	2006.4	n.a.	n.a.	2541.2±4513.5	n.a.	6.04±4.75	503.5±165.7	417.2±398.7	n.a.	n.a.	323.6±406.3	70.1±18.5	n.a.	n.a.	n.a.	[23]
		n.a.	n.a.	449-11750	n.a.	1.58-14.3	273-786	150-1212	n.a.	n.a.	66.3-1128	48.9-98.1	n.a.	n.a.	n.a.	
Old lianang River in Guiyu Town in China	2005.8, 2005.9	n.a.	n.a.	1070±1210	n.a.	4.09±3.92	324±143	230±169	n.a.	n.a.	181±156	n.a.	15.5±5.40	n.a.	n.a.	[22]
		n.a.	n.a.	125-4540	n.a.	n.d.-10.3	121-628	79.5-590	n.a.	n.a.	26.0-543	n.a.	9.51-24.1	n.a.	n.a.	

Notes: Mn in g kg^{-1}, others in mg kg^{-1}; "ND"indicates that the result is less than the detect limitation of the method; "n.a." presents that the values are not available.

Table S16 *Comparison of pollutant concentrations in surficial channel sediments in Lianjiang River Basin, and data on pollutant concentrations from other studies*

Rivers	Sampling date	Sb	Sn	Cu	Hg	Cd	Zn	Pb	Mo	Be	Ni	Cr	Co	As	Mn	References
Lianjiang River, China	2020.12	10.36±12.11	96.11±104.07	137.2±131.1	0.467±0.517	0.64±1.08	240.2±166.9	72.0±28.2	2.21±1.2	3.84±1.14	32.2±19	60.2±25.9	10.38±2.63	9.86±5.11	0.468±0.138	In this study
		0.79-41.70	6.99-405.28	13.4-477.5	0.072-2.063	0.03-4.30	67.5-669.5	26.1-115.0	0.56-4.09	1.84-5.77	12.1-66.1	25.5-106.2	6.01-15.24	3.81-19.6	0.203-0.703	
Lianjiang River. China	2019.11,2020.5, 2020.8	n.a.	n.a.	0-400	n.a.	0-0.83	0-558	0-132	n.a.	n.a.	0-69.0	n.a.	n.a.	n.a.	n.a.	[20]
Lianjiang River (Excluding Old Lianjiang River), China	2018.01	23.9	46.1	172.2	n.a.	0.53	296	87.2	n.a.	n.a.	41.6	69.1	10.53	n.a.	n.a.	[26]
Lianjiang River in China	2015.12, 2016.4, 2016.8	n.a.	n.a.	252±628	0.631	1.24±0.62	272±169	112±70.9	n.a.	n.a.	39.1	52.7±40.1	17.8	17.5±13.3	0.437	[28]
Tributaries, and mainstream of LR (downstream of Guiyu region) , China	2013.1	71.8	172	587	0.54	2.28	414	96	6.9	0.65	130	120	9.55	16.2	0.335	[21]
		8.10-245	16.6-375	43.6-3210	0.14-1.20	0.41-14.5	114-1070	32.4-256	2.23-15.6	0.27-1.19	26.1-427	29.8-379	3.22-23.4	7.77-32.7	0.260-0.399	
Lianjiang River, China	2005.10	n.a.	n.a.	620.9±478.9	0.493±0.197	0.556±0.089	n.a.	93.0±52.4	n.a.	n.a.	69.7±68.7	n.a.	n.a.	11.3±3.90	n.a.	[19]
		n.a.	n.a.	69.3-1264	0.311-0.930	0.437-0.724	n.a.	46.2-173	n.a.	n.a.	17.5-218	n.a.	n.a.	6.48-19.6	n.a.	
Feiyun River Basin, China	2021.9	4.89±5.85	n.a.	9.19±3.68	n.a.	1.03±1.83	323.53±505.55	41.88±27.5	4.14±2.45	n.a.	8.61±6.83	16.62±16.75	5.2±1.65	212.64±236.64	1.026±1.197	[18]
		0.59-21.56	n.a.	4.51-17.58	n.a.	0.16-6.62	74.41-1934.82	16.43-102.99	1.8-13.82	n.a.	0.56-28.25	ND-66.31	3.07-8.35	5.66-728.11	0.353-5.638	
Upstream Xijiang Basin, China	2014.6-2014.8	34.02±73.40	n.a.	27.07±10.71	0.31±0.15	4.92±4.42	416.51±563.32	113.09±139.45	n.a.	n.a.	28.03±6.39	57.58±12.21	n.a.	95.42±229.79	n.a.	[74]
		5.79-265.71	n.a.	16.71-57.41	0.14-0.61	1.12-17.50	125.15-2145.44	17.39-492.87	n.a.	n.a.	21.33-38.85	41.35-82.30	n.a.	18.58-824.26	n.a.	
Daqing River , China	2013.8-2013.9	n.a.	n.a.	73.91	n.a.	0.68	227.88	32.01	n.a.	n.a.	34.74	110.28	n.a.	n.a.	n.a.	[75]
		n.a.	n.a.	24.39-471	n.a.	0.21-1.43	64.51-811.73	7.74-72.06	n.a.	n.a.	17.39-61.93	57.14-288.38	n.a.	n.a.	n.a.	
Zhongba River , China	—	n.a.	n.a.	34.81±12.85	0.09±0.07	0.46±0.29	134.09±70.33	24.77±12.26	n.a.	n.a.	27.09±13.88	82.38±33.89	n.a.	5.65±1.82	n.a.	[76]
		n.a.	n.a.	18.49-61.22	0.05-0.27	0.25-1.11	66.39-258.64	14.69-49.04	n.a.	n.a.	13.06-52.16-	46.52-147.17	n.a.	3.69-9.41	n.a.	
Seomjin River . South Korea	2017-2018	n.a.	n.a.	27.0±7.2	0.052±0.014	0.26±0.07	153.0±31.6	29.2±5.0	n.a.	n.a.	20.9±4.9	47.0±9.7	n.a.	10.7±3.0	n.a.	[15]
Boseong River . South Korea	2017-2018	n.a.	n.a.	30.4±8.7	0.055±0.014	0.26±0.07	150.4±28.1	29.7±4.6	n.a.	n.a.	30.5±14.6	62.7±24.1	n.a.	13.9±6.0	n.a.	[15]
Streams in Northeast of Konya, Turkey	—	0.78	n.a.	48.8	0.57	0.10	73.66	13.2	0.55	n.a.	507	n.a.	52	18.8	n.a.	[77]
		0.10-4.20	n.a.	10.1-109	0.01-6.80	0.10-0.10	13.4-252.0	1.90-41.3	0.10-3.40	n.a.	52-2650	n.a.	13-117	5.70-75.3	n.a.	
Oued Rarai, north-western Tunisia	2016.3	32.1±45.2	n.a.	22.6±10.9	1.54±5.56	0.64±1.14	180±255	147±531	n.a.	n.a.	36.7±14.2	57.4±31.4	n.a.	85.8±170	n.a.	[13]
		0.02-297	n.a.	3.85-66.8	0.05-54.4	0.05-8.01	23.2-2610	2.90-5150	n.a.	n.a.	5.28-87.6	3.46-198	n.a.	0.50-1490	n.a.	
Amu Darya Basin , Central Asia	2011.10 2019.8	n.a.	n.a.	23.78±15.76	n.a.	0.36±0.30	68.73±29.12	18.61±8.71	n.a.	n.a.	28.37±19.19	59.67±34.11	10.40±3.30	n.a.	n.a.	[16]
		n.a.	n.a.	1.81-142.8	n.a.	0.04-1.81	20.87-20.87	3.28-52.84	n.a.	n.a.	1.46-228.2	2.88-384.1	2.40-22.56	n.a.	n.a.	

Notes: "n.a." indicates that the data are not available.

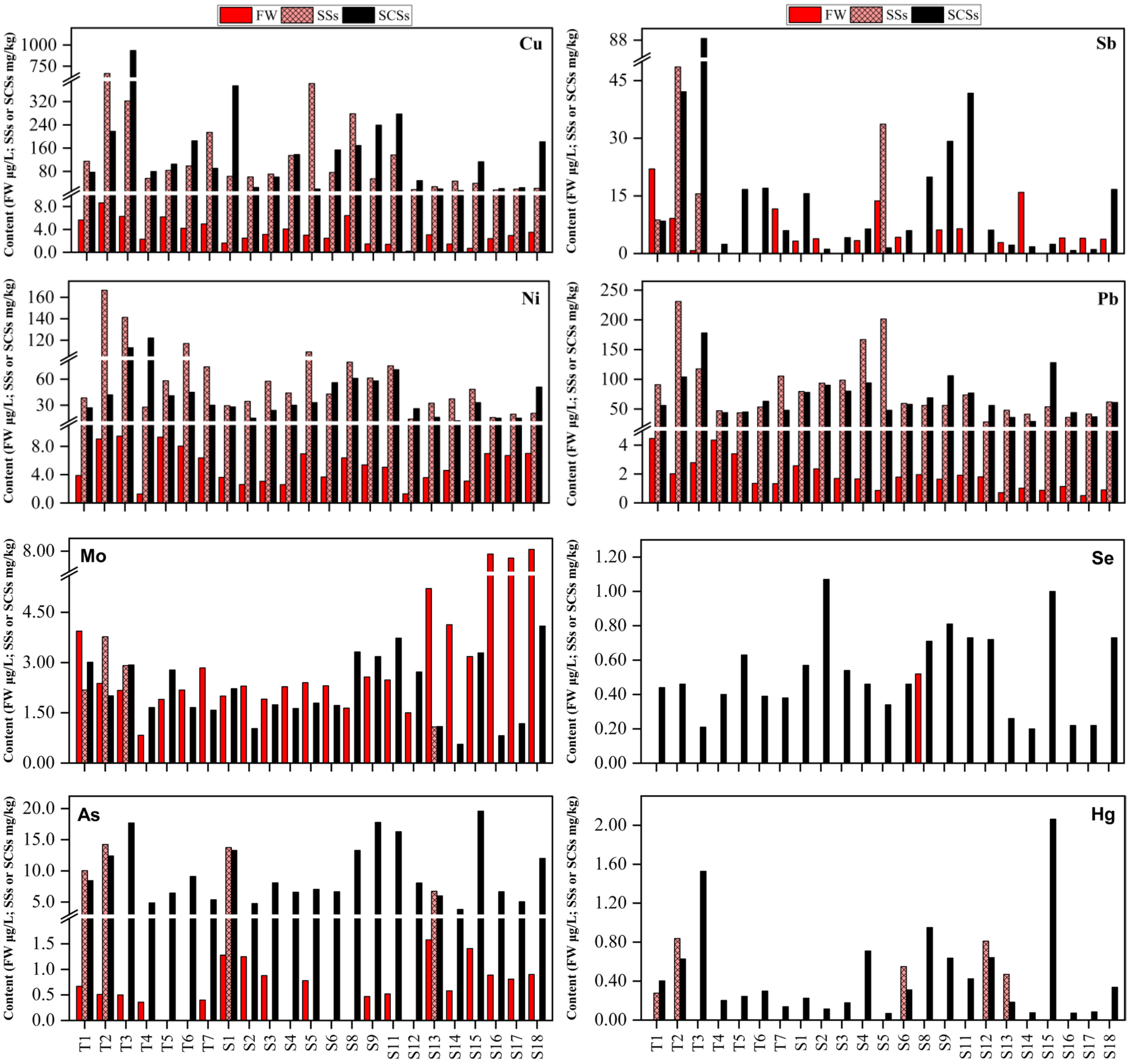

Figure S1 *The distribution profiles of of dissolved element concentrations in surface water (FW), dissolved elements in suspended solids (SSs), and pollutants in surficial channel sediments (SCSs)*

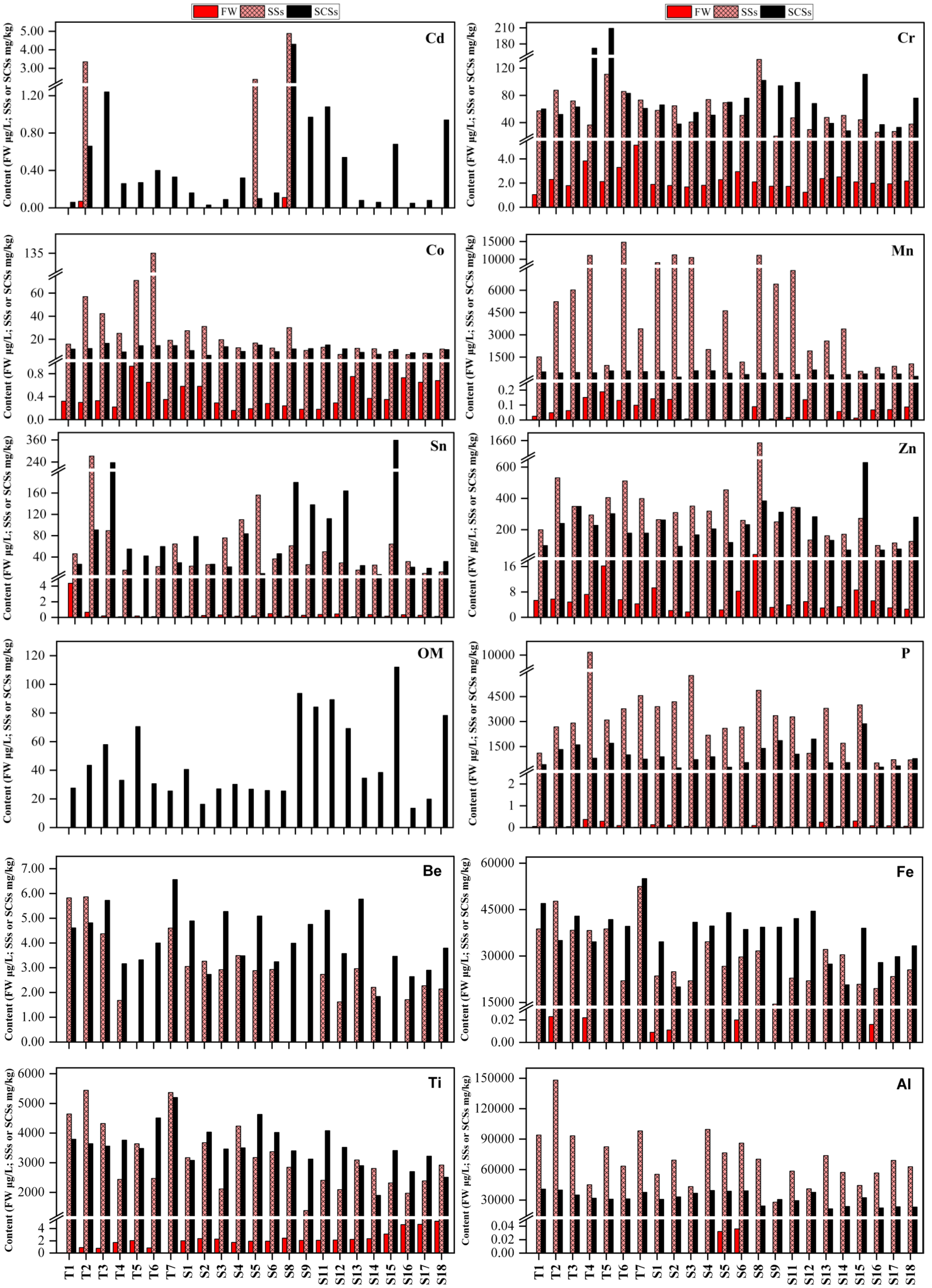

Figure S2 *The distribution profiles of of dissolved element concentrations in surface water (FW), dissolved elements in suspended solids (SSs), and pollutants in surficial channel sediments (SCSs)*

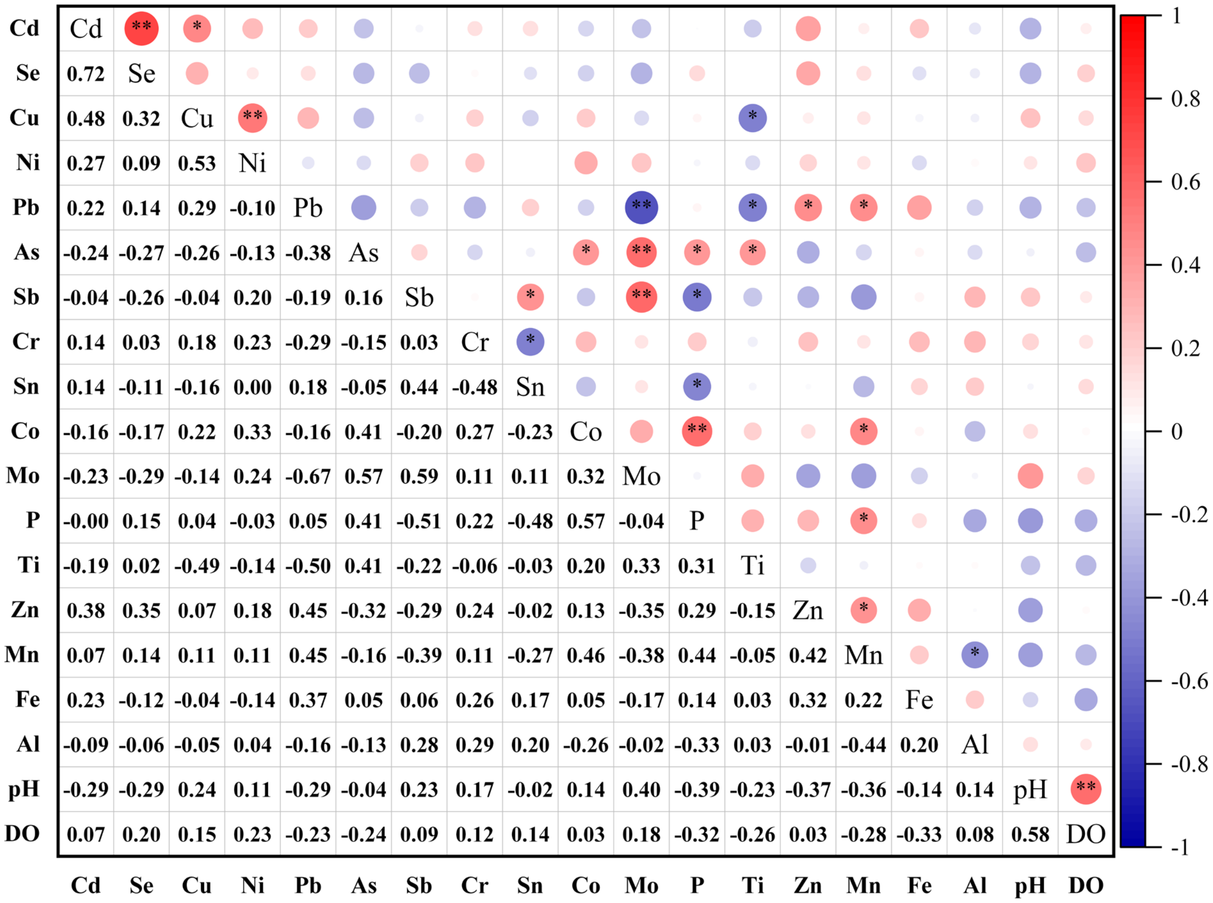

Notes: ** indicates that the elements are correlated at the 0.01 level of significance (two-tailed);

* indicates a 0.05 level of significance (two-tailed).

Figure S3 *Spearman correlation coefficient matrix for dissolved elemental concentrations, pH values, and dissolved oxygen (DO) contents in surface waters*

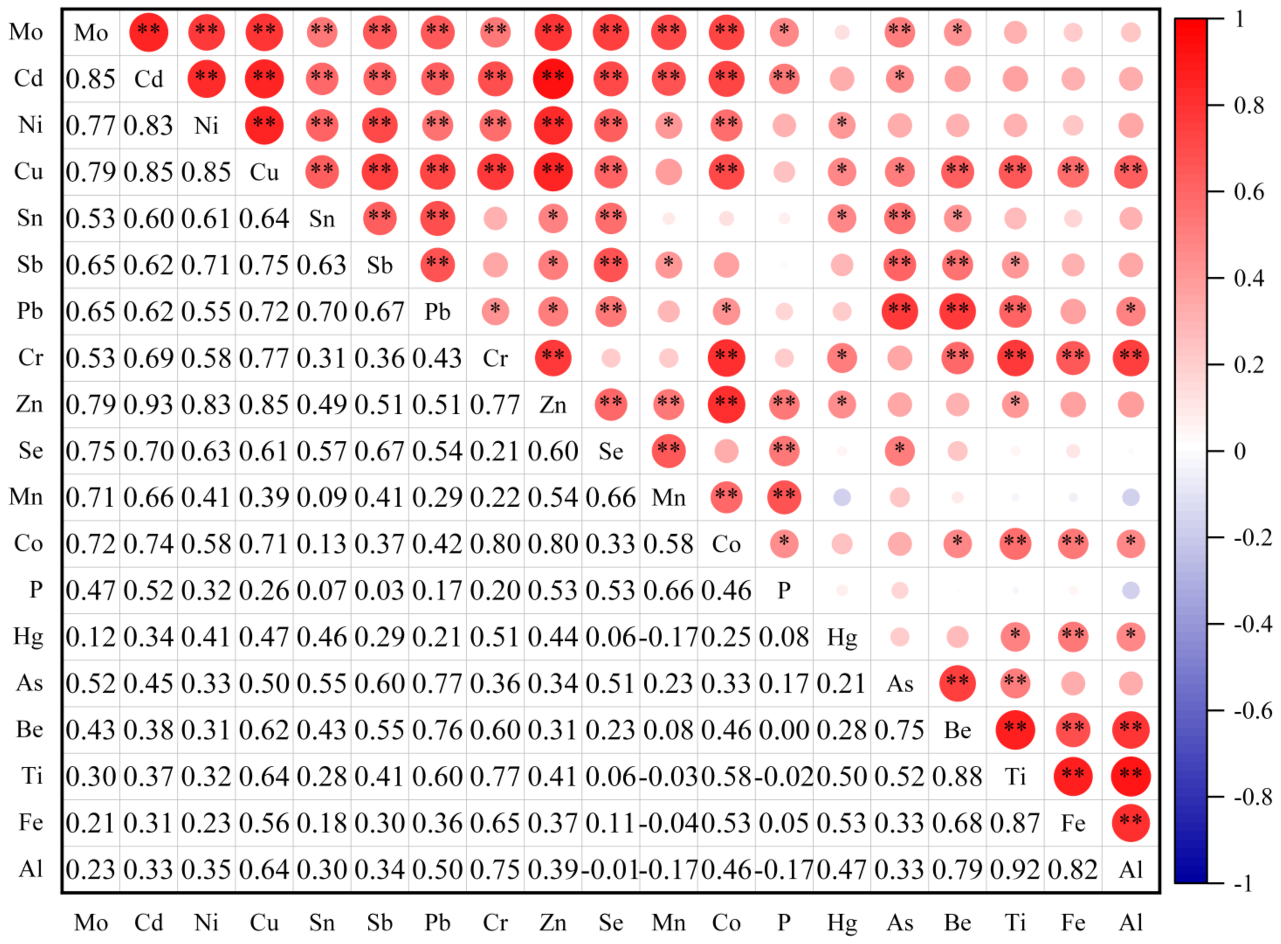

Notes: ** indicates that the elements are correlated at the 0.01 level of significance (two-tailed);

* indicates a 0.05 level of significance (two-tailed).

Figure S4 *Spearman correlation coefficient matrix for suspended solids-associated elemental concentrations in surface waters*

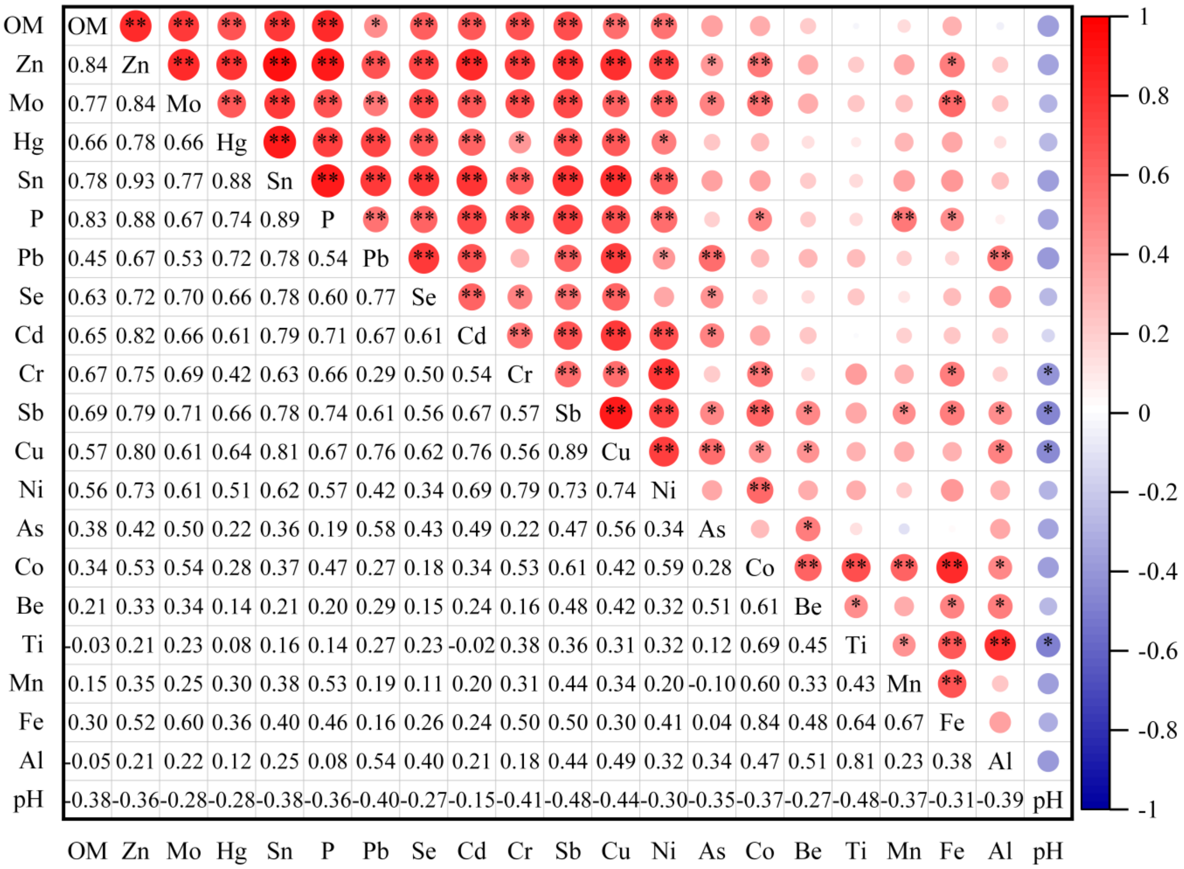

Notes: ** indicates that the elements are correlated at the 0.01 level of significance (two-tailed);

* indicates a 0.05 level of significance (two-tailed).

Figure S5 *Spearman correlation coefficient matrix for pollutants in surficial channel sediments*

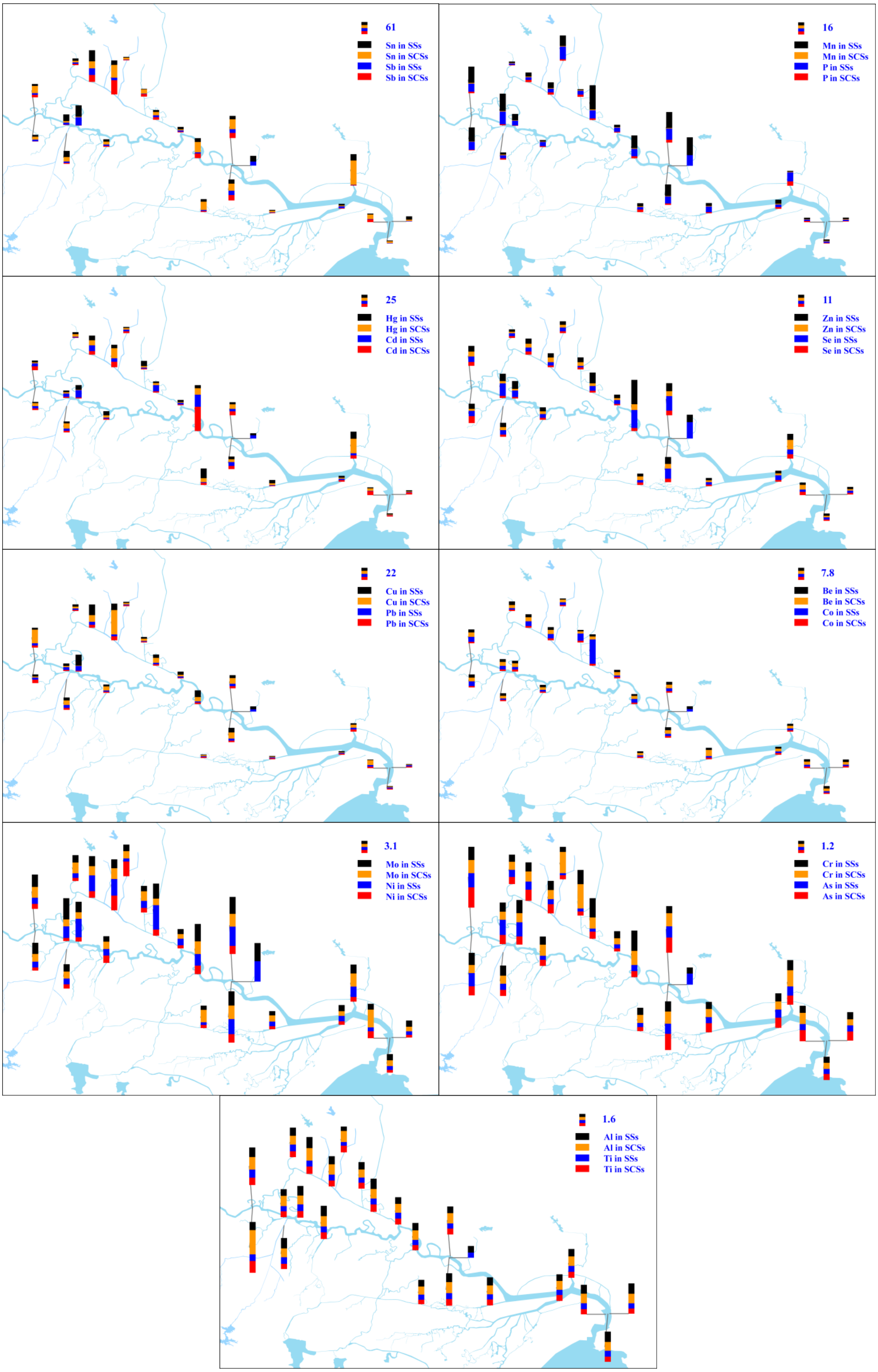

Figure S6 *Enrichment factors of elements detected in suspended solids (SSs) and surficial channel sediments (SCSs)*

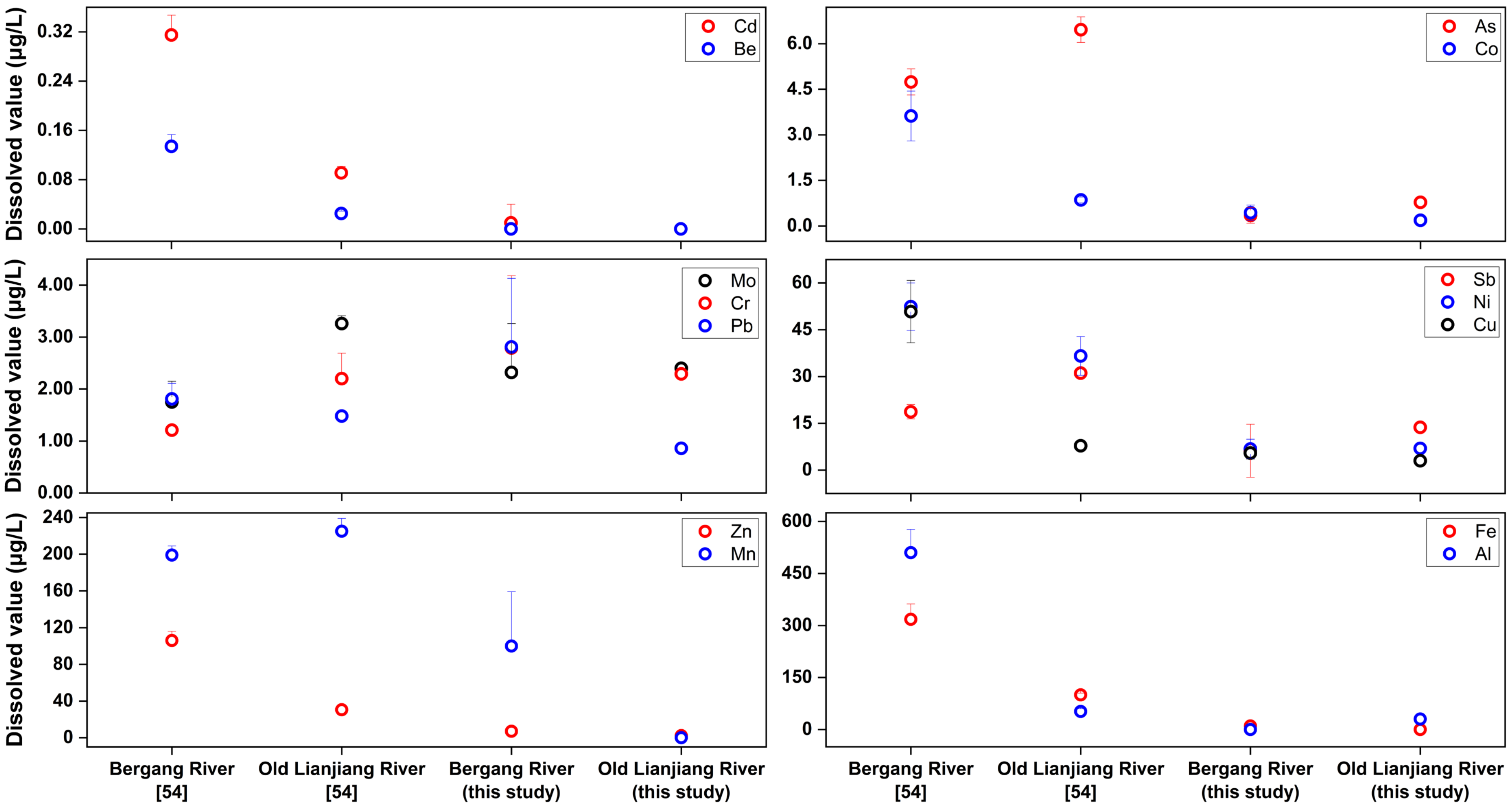

Figure S7 *Comparison of dissolved elemental values in surface water in Guiyu Town*

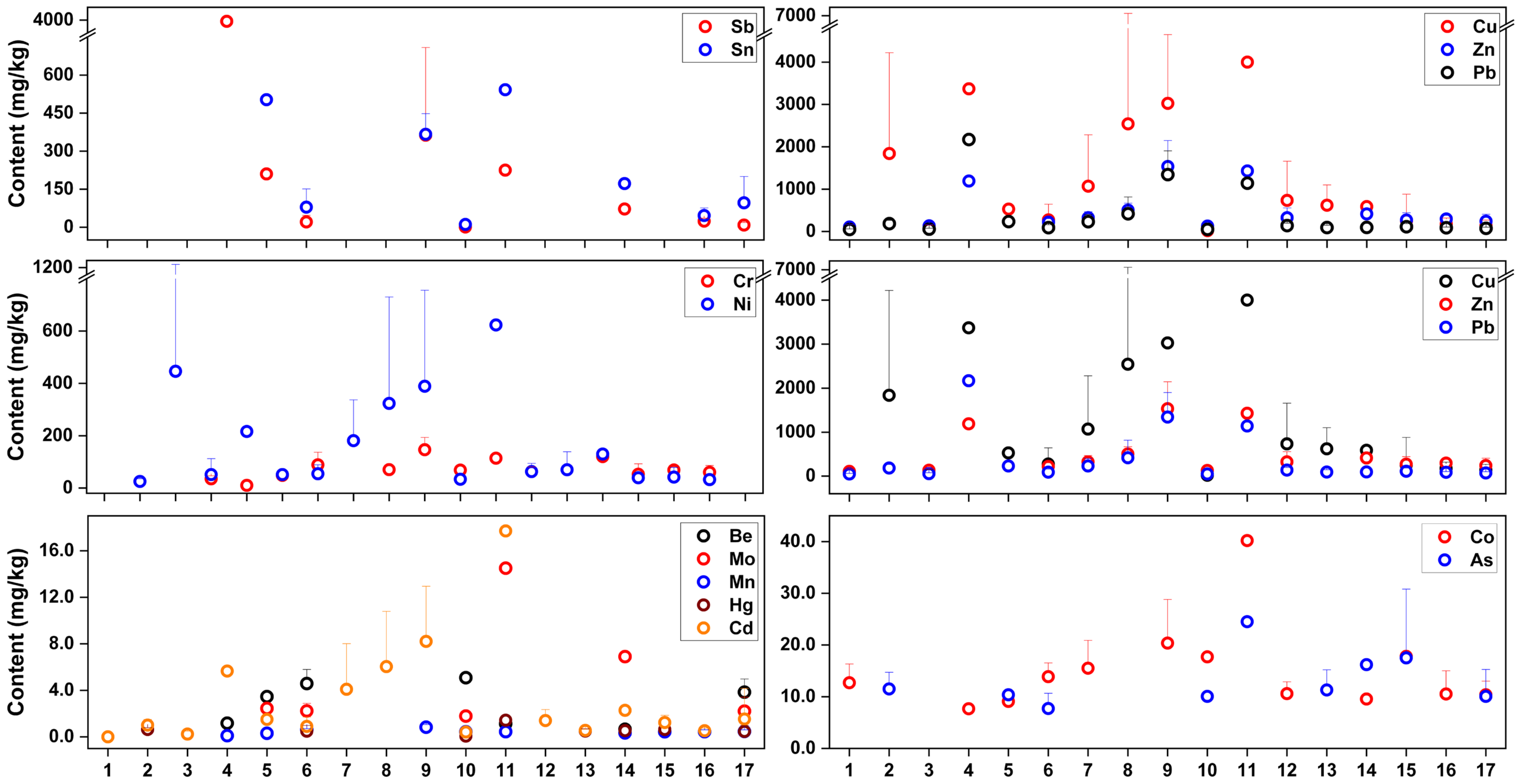

Notes: Mn in g/kg, others in mg/kg;
Bergang River: 1: Wong et al.[22]; 2: Guo et al.[19]; 3: Mao et al.[21]; 4: Quan et al.[24]; 5: Wu et al.[25]; 6: this study;
Old Lianjiang River: 7: Wong et al.[22]; 8: Mao et al.[21]; 9: Du et al.[26]; 10: this study;
Rivers in Guiyu Town: 11: Mao et al.[21]; 12: Xiong et al.[27];
Lianjiang River: 13: Guo et al.[19]; 14: Mao et al.[21]; 15: Shi [28]; 16: Du et al.[26]; 17: this study.

Figure S8 *Comparison of metal values in surficial channel sediments in the Lianjiang River Basin*
